# Insights into the key interactions between human protein phosphatase 5 and cantharidin using molecular dynamics and site-directed mutagenesis bioassays

**DOI:** 10.1038/srep12359

**Published:** 2015-07-20

**Authors:** Ji-Yuan Liu, Xi-En Chen, Ya-Lin Zhang

**Affiliations:** 1 Key Laboratory of Plant Protection Resources & Pest Management of the Ministry of Education, Northwest A&F University, Yangling 712100, Shaanxi, China

## Abstract

Serine/threonine protein phosphatase 5 (PP5) is a promising novel target for anticancer therapies. This work aims to uncover the key interactions at the atomic level between PP5 and three inhibitors (cantharidin, norcantharidin and endothall). We found that, unlike previous report, Arg 100 contributes less to PP5-inhibitor binding, and the residues His 69, Asn 128, His 129, Arg 225, His 252 and Arg 250 are of importance to PP5-inhibitor binding. The hydrophobic interactions established between the residues Val 254, Phe 271 and Tyr 276, especially Glu 253, are very important to enhance the inhibitive interaction. We suggested that, to increase the inhibitory activity, the interactions of inhibitor with three negatively charged unfavorable interaction residues, Asp 99, Glu 130 and Asp 213, should be avoided. However, the interactions of inhibitor with favorable interaction residue Arg 250 could enhance the inhibitory activity. The Manganese ion 2 (MN2) unfavorably contribute to the total interaction free energies. The coordination between MN2 and chemical group of inhibitor should be eliminated. This work provides insight into how cantharidin and its analogs bind to PP5c at the atomic level and will facilitate modification of cantharidin-like chemicals to rationally develop more specific and less cytotoxic anti-cancer drugs.

The reversible phosphorylation of serine and threonine residues on proteins executed by kinases and phosphatases plays a crucial role in regulating many biological responses^1^. These two processes constitute a major form of signaling and an essential mechanism of regulation in all living organisms. In eukaryotic cells, phosphorylation mainly occurs on the residues serine, threonine, and tyrosine, of which serine is the predominant target^2^. Protein phosphatase 5 (PP5) is a member of the family of protein serine/threonine phosphatases (PPP) which also contains PP1, PP2A, PP2B, PP2C, PP4, PP6 and PP7. It regulates cellular proliferation, differentiation, migration, apoptosis, and DNA damage repair. In particular, PP5 plays important roles in regulating the dynamic phosphorylation of many signaling components including p53, apoptosis signal-regulating kinase 1 (ASK-1), and mitogen-activated protein kinase (MAPK)^3^,^4^,^5^,^6^. PP5 is expressed ubiquitously in all mammalian tissues examined and is highly conserved among eukaryotes. The high PP5 protein levels are associated with the development of the liver, and are observed in human cancers where the constitutive overexpression of PP5 aids tumor progression in mouse models of tumor development^7^. Moreover, elevated PP5 expression levels were also observed in human breast cancer^8^. Taken together, these studies have indicated that PP5 is a promising novel target for anti-cancer therapies^9^,^10^.

Cantharidin is an active constituent of the bodies of dried blister beetles and is a strong inhibitor for protein phosphatase PP1, PP2A, PP4 and PP5 that plays an important role in the control of the cell cycle, apoptosis, and cell-fate determination. The inhibiting activity of cantharidin against PP5 has been examined at the nanomolar level with a relative IC_50_ value of 600 nM^11^,^12^,^13^. Cantharidin and its demethylated form, norcantharidin, exhibited strong *in vitro* and *in vivo* antitumor activity against various types of cancer cells, especially hepatocellular carcinoma cells^14^. The underlying mechanisms of antitumor activity mainly involves DNA damage and apoptosis through the inhibition of protein phosphatases^15^,^16^. However, due to the severe side-effects of cantharidin on the gastrointestinal tract, kidney, and ureter, the clinical application of cantharidin is limited and has prevented approval by the Food and Drug Administration^17^. Norcantharidin is less cytotoxic than cantharidin and has been used to treat human cancers in China since 1984^18^. Through extensive efforts, less cytotoxic derivatives have been developed. One is the novel pharmacological PP2A inhibitor LB100, a candidate agent currently entering Phase I clinical trials. Pharmacological inhibition of PP2A produces anti-tumor activity against many human cancer types highlighting PP2A as an attractive target for the development of novel anti-cancer drugs with an emphasis on cantharidin and norcantharidin analogues^19^.

The catalytic domain of PP5 (PP5c) shares 35–45% sequence identity with the catalytic domains of other members of the PPP family. Actually, the superimposition root-mean-square deviation (RMSD) with the structures of the catalytic domains between the available X-ray structure of PP5c (PDB ID:1WAO) and PP2A (PDB ID:2IE4) is less than 1.0 Å, and the RMSD is even smaller if the superimposition is limited to the active site^20^,^21^. It is conceivable that the rational design of more specific, possibly less cytotoxic, cantharidin-like drugs can be facilitated by understanding the related X-ray complex structures and study of the mechanism for the protein-inhibitor interactions. The high-resolution crystal structures of PP5c soaked with the corresponding dicarboxylic acid derivatives of the cantharidin and norcantharidin have been reported, which provide a suitable general model to investigate the structural basis for the inhibition of PPP by cantharidin and its derivatives^22^. Furthermore, understanding the mechanism of protein-inhibitor interactions provides the molecular basis for designing new compounds with higher potency^23^. The active center of PP5 contains two manganese ions and a total of ten catalytic residues. Previously studied X-ray structures combined with theoretical studies on the reaction mechanism of PP5c provide some clues that Arg 100, Asn 128, His 129 and Arg 225 are key residues for binding the phosphate moiety of substrates^24^,^25^, and two manganese ions(M1, M2) , Arg 100, Asn 128, His 129, and Arg 225, together with the interactions of water (W1) with M1, M2, and His 252 might be involved in inhibitor binding^10^. So far, the key interactions at the atomic level between the cantharidin and PP5c are still not clear. Molecular dynamics (MD) studies of proteins can provide deep insights into the structure and dynamics of proteins at the atomic level. The MD properties of a protein have a profound effect upon its functional behavior, and characterizing protein internal motions is therefore complementary and synergic to structural studies^26^,^27^.

In this study, we perform molecular dynamics (MD) simulations and site-directed mutagenesis to illustrate how cantharidin and its analogs bind to PP5c at an atomic level. This will be significant for chemical modifications in the structure of cantharidin, norcantharidin and endothall, facilitating rational design of more specific, possibly less cytotoxic, cantharidin-like drugs.

## Results and discussion

### MD simulations

**Figure 1:**
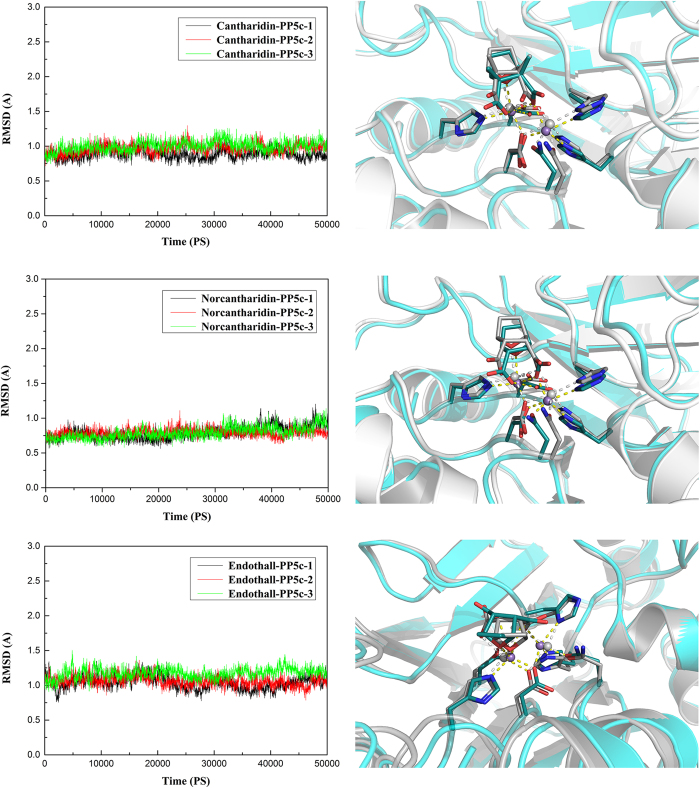
RMSD values for the three complexes structures monitored along three individual 50 ns production phase MD simulations for the whole proteins (left), and the last snapshot retrieved from the 50 ns production phase MD simulations compared with the crystal structures (right). The last snapshot conformations as obtained from MD simulations are in deep cyan; the conformations as obtained from the crystal structure are in gray. The manganese ions are presented as slate spheres. Cantharidin, norcantharidin and endothall are presented with the stick model. Residues which ligated the manganese ions are also presented with the stick model. Yellow and white dashed line represented the coordination bond in last snapshot and crystal structure, respectively.

In order to validate the parameters concerning the two manganese coordination center of the three systems shown in Table S1 and Figure S4, we submitted all three systems (Figure S2) to perform three individual 50 ns MD simulations. The thrice root-mean-square deviation (RMSD) of each system is illustrated in Fig. 1. The conformation of the three complexes simultaneously achieved equilibrium at ~10 ns with RMSD average value around 0.95 Å, 0.79 Å and 1.09 Å, respectively, suggesting that the deviations were very small and the structures were stable during the MD simulation. The last snapshot retrieved from the MD trajectory of each system overlap well with the reference crystal structures, indicating our parameters were able to maintain the geometry of the manganese coordination center very well during the entire MD simulation (Fig. 1).

**Figure 2:**
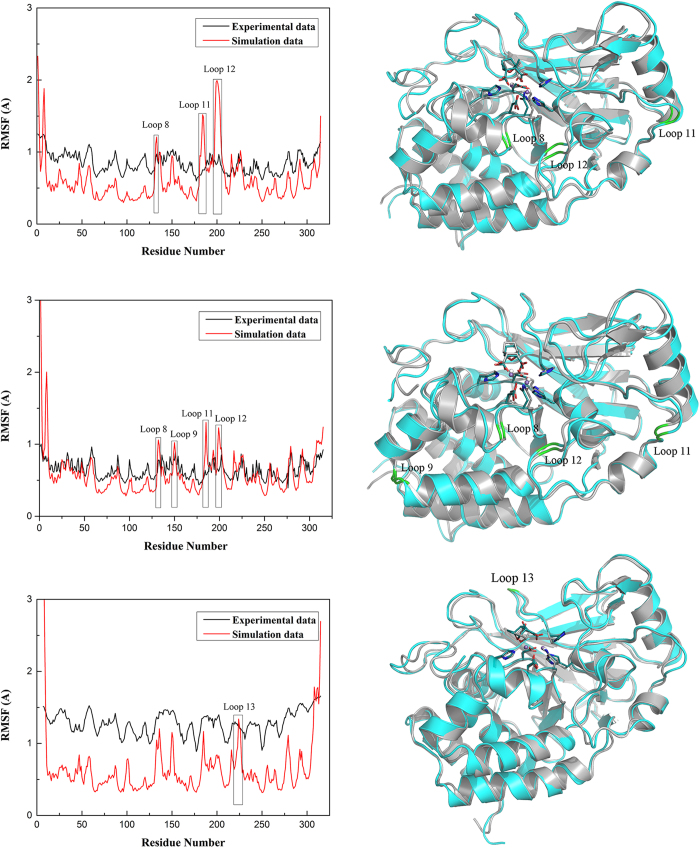
Residue fluctuations obtained by average residual fluctuations over 50 ns MD simulations are displayed in red. Experimental results calculated from B factors of crystal structures of PP5c complexes (PDB code: 3H63; 3H61; 3H64) are shown in black (left). Superimpositions of the averaged conformation of the cantharidin, norcantharidin and endothall in the PP5c along MD simulations on those obtained from the crystal structures. The conformations obtained from MD simulations are in deep cyan; the conformations obtained from the crystal structure are in gray (right).

The structure of PP5c is composed of a 12-stranded central β sheet surrounded by 7 α helices and linked by loops of varying length (Figure S5). In order to demonstrate the fluctuations of residues with the conformational transition, the root-mean-square fluctuation (RMSF) profiles based on the thrice 50 ns MD simulation trajectories of each system were further analyzed. The differences in RMSF between the MD simulations and the B-factor of the corresponding crystal structures (*B* = 8*π*^2^*RMSF^2^/3) are displayed in Fig. 2. Comparing with RMSFs, the Cantharidin-PP5c and Norcantharidin-PP5c complexes share very similar RMSF profiles, and few differences were observed for the Endothall-PP5c complex between the MD simulations data and experimental data. As a whole, α helices and β sheets exhibit a rigid behavior in these enzymes, and the loops represent relatively larger fluctuations. The loop 12 in Cantharidin-PP5c complex is most flexible with an RMSF difference less than 1 Å compared to the other two complexes. This suggests that loop 12 is freely exposed to the solvent to a larger degree. The loop 11 and loop 8 in the Cantharidin-PP5c complex, and the loop 8, loop 9, loop 11 and loop 12 in the Norcantharidin-PP5c complex have small RMSF differences of less than 0.5 Å, especially loop 13 from the Endothall-PP5c complex which has a lower RMSF difference of less than 0.1 Å. According to Fig. 2, the averaged structures of three ligands and the manganese center (manganese ions and the residues coordinated to them) derived from the MD simulations resemble their conformations in the crystal structures, with the RMSD (backbone-superimposed) below 0.63 Å. These findings indicate that the results of our MD simulation could capture the main behavior in the atomic movement of the three complexes.

To further verify the accuracy of the force field parameters, we calculated the distances of the manganese coordination bond over 50 ns. These distance values are listed in Table S2, and their time dependences are plotted in Figure S6. The bond distances between the manganese ion and the atoms ligated to it fluctuate only slightly during the course of MD simulations in three complexes, and their average values show well agreement with the X-ray structures except two pairs of atoms (Asp 96@OD2-MN1; Endothall@O2-MN2) are longer than 0.4 Å in the Endothall-PP5c complex. The relative longer distances within the two pairs of atoms might due to the thermal motion. The comparative results of the vibrational frequencies calculated from normal-mode analysis to those from the WB97XD/6-31G** level calculation are illustrated in Figure S7. This match between the MM normal mode and the QM calculated frequencies is almost perfect despite the deviation being above 3000 cm^−1^.These findings demonstrated that our force field parameters can accurately reproduce the correct three dimensional structures for the manganese coordination spheres and can be applied to the molecular dynamics simulation of the other members of the PPP family.

### Binding free energy

To further validate the correspondence between binding free energy calculation and our experimental results, we employed the MM-PBSA approach to calculate absolute binding free energy to compare the binding affinities of cantharidin, norcantharidin and endothall in the binding pockets of PP5c^28^, and to gain insight into differences in binding. The binding affinities (ΔG_bind-calc_) and the energetic components for the three complexes are summarized in Table S3 based on the last 10 ns production three trajectories. According to our results, ΔG_bind-calc_ of cantharidin, norcantharidin and endothall bound with PP5c are −16.67, −7.36 and −13.91 kcal/mol, respectively. The rank of the binding affinity level predicted by ΔG_bind-calc_ values for the three inhibitors is: Cantharidin > Endothall > Norcantharidin. This is in good agreement with the IC_50_ values of our following enzyme assays, and supports the reliability of our force field parameters and MD simulations. Although the entropic contributions to the binding free energy are not quantitatively equal, the rank of the binding affinities of the three complexes could be qualitative.

As shown, the major favorable contributor to the three inhibitors is the electrostatic energy term (ΔE_ele_), whereas the electrostatic contribution to the solvation free energy term (ΔG_PB_) is unfavorable to the ligands binding. Large differences are observed in the van der Waals contributions (ΔE_vdW_). The van der Waals energies (ΔE_vdW_) for the Cantharidin-PP5c and Norcantharidin-PP5c complexes are 5.06 and 29.38 kcal/mol, respectively, which are smaller than that of the Endothall-PP5c complex (−1.31 kcal/mol). Our findings suggest that both the electrostatic energy (ΔE_ele_) and the van der Waals energy (ΔE_vdW_) determine the binding affinities of the ligands to the active pocket of PP5c.

### Spectrum of free energy decomposition for each residue contribution

**Figure 3:**
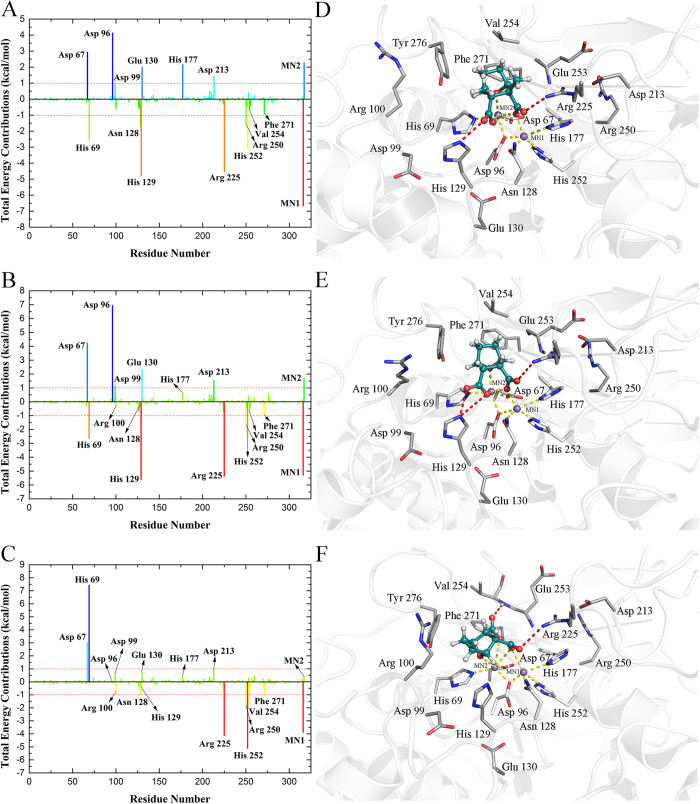
Residue-ligand interaction spectrum of (**A**) the Cantharidin-PP5c complex, (**B**) the Norcantharidin-PP5c complex and (**C**) the Endothall-PP5c complex according to the MM-PBSA method. The x-axis denotes the residue number of the PP5c and the y-axis denotes the total interaction free energy contribution for each residue. The key interactions and H bond patterns at the active site observed during MD simulations of cantharidin (**D**), norcantharidin (**E**) and endothall (**F**). The manganese ions are presented as slate spheres. Cantharidin, norcantharidin and endothall are presented with the stick-and-sphere model. Color code: deep cyan, C; red, O; white, H. Key residues are presented with the stick model. Color code: gray, C; red, O; blue, N; white, H; yellow dashed line, coordination bond; red dashed line, H bond.

The key residues contributing more than 1 kcal/mol to the total interaction free energies are illustrated in Fig. 3 (left). The positive charged Arg 100 acts as the gatekeeper and the hydrophobic pocket defined by the side chains of Val 254, Phe 271, and Tyr 276 in the active site of PP5c as mentioned in the previous study^22^, the unfavorable interaction residues and two manganese ions were also involved in the decomposition of interaction free energies on a per-residue level, and the detailed energetic components are listed in Table S4. Figure 3A shows that the residues of the Cantharidin-PP5c complex in the binding surface contribute more than 1 kcal/mol to the total interaction free energies including His 69, Asn 128, His 129, Arg 225, His 252 and Arg 250. According to the Table S4 and Fig. 3D, the sidechain atoms of the residues His 69, Asn 128 and His 252 contribute remarkable electrostatic energies to the total interaction free energies, which is in accordance with our calculation of RESP charge fitting (Figure S4). The prominent contribution to the total interaction free energies of the gatekeeper Arg 100 and Arg 225 are mainly from electrostatic energies, especially the residue Arg 225 that achieved −4.54 kcal/mol. This is a consequence of the hydrogen bond interaction between Arg 225 and cantharidin (Fig. 3D),the H-bond having a distance of 2.82 Å between the NH1 atom (HH11) of the residue Arg 225 sidechain and the carbonyl oxygen atom (O5) derived from the right carboxylate moiety of the cantharidin exhibits a high H-bond occupancy rate (97.09%) (Table 1).The hydrophobic cavity was consistent with the side chains of Val 254, Phe 271, and Tyr 276. The backbone atoms of Glu 253 primarily contribute the Van der Waals energy to the binding (Fig. 3D). The residues Val 254, Phe 271 and Tyr 276 combine as a “claw” in the hydrophobic cavity to make significant favorable contributions to the total interaction free energies. Noticeably, the residue His 129 possesses a negative energy with a high value, −4.79 kcal/mol. It is not surprising since the sidechain atoms of the residue His 129 can form the important hydrogen bond with cantharidin (Fig. 3D), the carbonyl oxygen atom (O4) derived from the left carboxylate moiety of the cantharidin and the NE2 atom (HE2) of the residue His 129 sidechain form an H-bond with a distance of 2.77 Å between atoms revealing a medium H-bond occupancy rate (60.03%) (Table 1).Intriguingly, the residue Arg 250 is far from the active center with the distance of approximately 10 Å (Fig. 3D), however, its contribution is more than 1 kcal/mol. These findings were also observed both in the norcantharidin-PP5c and Endothall-PP5c complexes. On the contrary, the residues Asp 67, Asp 96, Asp99, Glu 130, His 177 and Asp 213 offer unfavorable interaction free energies. This is a consequence of their large unfavorable electrostatic energies derived from sidechain, resulted from the strong negative charge repulsion established between these residues and cantharidin. It is worth noting that Asp 99, Glu 130, and Asp 213 belonging to uncoordinated residues, suggesting that mutation of these three residues might increase the inhibitory activity of PP5c inhibitors. Interestingly, two manganese ions coordinated with cantharidin contribute high electrostatic energies (−50.87 and −54.36 kcal/mol, respectively), however, only MN1 provides favorable interaction free energies benefiting for cantharidin binding. Because the stronger coordination interaction can produce a stronger repulsion of the Van der Waals decreasing the Van der Waals energy contribution, this inference agrees with MN2 contributes higher unfavorable Van der Waals energy.

Figure 3B and Table S4 depicts how the interaction spectrum of the Norcantharidin-PP5c complex has very similar features to that of the Cantharidin-PP5c complex. The residues His 69, His 129, Arg 225, Arg 250 and His 252 are mainly responsible for norcantharidin binding to PP5c. Especially the residues His 129 and Arg 225 both contribute more than 5 kcal/mol in total interaction energy to the norcantharidin binding, which is in accordance with the H-bond networks established between these two residues and PP5c in Norcantharidin-PP5c complex. The NH1 (HH11) atom of Arg 225 makes an H-bond with the carbonyl oxygen atom (O5) of the right carboxylate moiety of the norcantharidin, as revealed by an H-bond occupancy rate of 93.41% and the distance 2.78 Å between atoms. Two H-bonds are also formed between the sidechain of His 129 and the left carboxylate moiety of the norcantharidin. The NE2 atom (HE2) of His 129 forms H-bonds with the carbonyl oxygen atom (O3) and hydroxyl oxygen atom (O4) of the left carboxylate moiety of the norcantharidin, exhibiting H-bond occupancy rates of 70.47% and 19.05%, respectively (Table 1). Differing from the Cantharidin-PP5c complex, the residue Asn 128 contributes less than 1 kcal/mol energy (−0.23 kcal/mol) to the binding of the norcantharidin. Moreover, the residue Tyr 276 located in the hydrophobic cavity dominating the favorable electrostatic and Van der Waals energy contributions, the total interaction free energy is positive which is mainly caused by the unfavorable polar solvation energy (Fig. 3E). The residue His 177 offer unfavorable interaction free energy less than 1 kcal/mol (0.65 kcal/mol) compared with that of the Cantharidin-PP5c complex, this is consistent with its lower RESP charge (0.0055 |e|) (Figure S4). It could not form a strong electrostatic repulsion as like in the Cantharidin-PP5c complex to the negative charged carboxylate group (−1.32 |e|) derived from norcantharidin.

Due to the ca. 120° rotation of endothall when compared to the structure of cantharidin and norcantharidin, the interaction spectrum of the Endothall-PP5c complex shows many differences with the Cantharidin-PP5c and Norcantharidin-PP5c complexes. In Fig. 3C, the residues Asn 128, Arg 225, His 252, Arg 250, Val 254 and Phe 271 of the Endothall-PP5c complex in the binding surface make significant favorable contributions (>1 kcal/mol) to the total interaction free energies. In particular, Arg 225 exerts a 4 kcal/mol enhancement of the energy favorable to the total interaction free energies, which is consistent with the H-bonding networks formed in this complex. The H-bond is formed between the NH1 atom (HH11) of the residue Arg 225 sidechain and the carbonyl oxygen atom (O3) of the right carboxylate moiety of the endothall, revealing a medium H-bond occupancy rate of 63.22% (Table 1). In contrast, the residues Asp 67 and His 69 offer unfavorable energy (+3.04 and +7.44 kcal/mol, respectively). By comparison, the residue Asn 128 in the interaction spectrum of the Endothall-PP5c complex contributes more than 1 kcal/mol to the total interaction free energies. This is a consequence of the much closer distance between the carboxylate group of the endothall and the amide group of the residue Asn 128 sidechain in the Endothall-PP5c complex (Fig. 3F). The differences were also found in the hydrophobic cavity of the Endothall-PP5c complex. For the residue Val 254, the electrostatic energy derived from its side chain atoms is the major force that drives the binding of endothall to PP5c, which corresponds with the hydrogen bonds formed in this complex (Fig. 3F), due to the rotation of endothall, an additional H-bond is formed between the residue Val 254 and the endothall. The H-bond between the hydroxyl oxygen atom (O5) of the left carboxylate moiety of the Endothall and the N atom (H) of the residue Val 254 backbone is maintained with the H-bond occupancy rate of 69.84%, and the distance between the O5 and H atom is 2.92 Å (Table 1). Interestingly, differing from Cantharidin-PP5c and Norcanthadirin-PP5c complex, the residue Asp 96 in Endothall-PP5c complex mildly contribute to the total interaction free energy. The residue Asp 99, Glu 130, Asp 213 and manganese ion MN2 offer unfavorable energy to the total interaction energies less than 1 kcal/mol (Fig. 3C).

### Alanine mutations reveal the key residues involved in inhibitor binding

According to the interaction free energies decomposition, related residues in three complexes are presented in Figure S8. For clarity, the residues with the most favorable or unfavorable sidechain energy contribution of more than 1 kcal/mol are labeled in the figure. The labeled residues in the three complexes were further considered to perform the computational alanine scanning mutagenesis (ASM) besides the coordination residues, i.e., Asp 67, His 69, Asp 96, Asn128, His 177 and His 252. The residue Arg 100 was also selected to mutate to alanine due to the fact that its electrostatic energy contributes significantly to the total interaction free energy in the three complexes (Table S4). The favorable interaction residue His 129 and the unfavorable interaction residues Asp 99, Glu 130 and Asp 213 in the Endothall-PP5c complex was also selected to perform ASM in order to compare the differences with the other two complexes (Table S4). The ASM results for the selected residues are given in Table 2. The positive values suggest that the mutations disfavor the binding of the inhibitors. On the other hand, a mutation resulting in a more negative ΔΔG_bind_ is considered to be more favorable. Interestingly, we found that the PP5c-inhibitor interactions in three complexes were enhanced when Asp 99, Glu 130 and Asp 213 were mutated to Ala, especially E130A mutation, whose total interaction free energy was changed by more than 2.5 kcal/mol. The most likely reason is that Asp 99, Glu 130 and Asp 213 are negatively charged, and the dibasic carboxylic group in the inhibitors is also negatively charged, these three negatively charged residues showed potent electrostatic repulsion to the negative charged groups of inhibitors. The ASM mutations clearly indicate that the mutation of Arg 100 has little impact on the three inhibitors binding with the difference of binding free energy as it is ~1 kcal/mol in all cases. The most significant impacts on the binding of cantharidin and norcantharidin were affected by the His129Ala (H129A) mutation with the changes of 14.68 and 11.36 kcal/mol, respectively. On the contrary, the mutation H129A in the Endothall-PP5c complex cannot make the binding free energy drop dramatically with a change of only 3.42 kcal/mol. The relative low ΔΔG_bind-calc_ is highly likely due to the ca. 120° rotation of endothall in the Endothall-PP5c complex which makes the H-bond contribution of His 129 vanished, however, this residue is still regarded as a hot-spot. The mutation of Arg 225 has a greater impact on the binding of the three inhibitors causing the binding free energies to shift unfavorably by 10.38, 10.45 and 12.26 kcal/mol, respectively. Mutation of the residue Arg 250 to alanine resulted in a larger change to the binding free energy by more than 2.0 kcal/mol in the three complexes. The Val254Ala mutation in the Endothall-PP5c complex produced an impact of 1.82 kcal/mol on endothall binding. On the basis of the computed free energy changes for Ala mutations, His 129 in the Cantharidin-PP5c and Norcantharidin-PP5c complex, and Arg 225 in the three complexes are classified as hot-spots, respectively. Previous study only suggested that Arg 100 is a key residue involving in the binding of PP5c with canthridin and its analogs^22^. These hot-spots were then considered prime candidates for performing experimental site-directed mutagenesis. Furthermore, in order to investigate why the computational ΔΔG_bind_ (ΔΔ_Gbind-calc_) of mutation H129A in the Endothall-PP5c complex is much lower than that of in the other two complexes, and to further assess the role of residue Arg 100 in the three complexes, these two residues were also included in the experimental site-directed mutagenesis.

A PCR-based site-directed mutagenesis approach was used to change Arg 100 to Ala (R100A), His 129 to Ala (H129A), and Arg 225 to Ala (R225A). All recombinant enzymes were soluble, expressed in *E. coli* and purified to homogeneity (>90% pure) (Figure S9). The three mutants at the phosphatase domain significantly affected the characterization of recombinant HuPP5. Kinetic parameters are presented in Table S6. Cantharidin, norcantharidin and endothall showed a potent inhibitory effect on recombinant wild-type HuPP5 with their IC_50_ values calculated as 0.82 μM, 4.18 μM, and 3.64 μM, respectively (Table S7). However, their inhibitory activity was suppressed against the three individually site-directed mutagenesis HuPP5: R100A, H129A, and R225A (Table S7). H129A appears to be most unfavorable to the binding of these inhibitors due to the highest IC_50_ values (>10 mM). Norcantharidin and endothall exhibited similar inhibitory effects on individual proteins, except for acting on R225A. The IC_50_ value of endothall on R225A was much higher than that of cantharidin (48.54-fold) and norcantharidin (6.47-fold), indicating that Arg 225 might be the key residue involved in endothall binding.

In order to further verify the key residues as hot-spots, the experimental ΔΔG_bind_ (ΔΔG_bind-exp_) for both the wild-type (WT) and mutants have been calculated (Table 2). As shown in Table 2, the ΔΔG_bind-exp_ of R100A in all three complexes were in close agreement with those determined by computational site-directed mutagenesis. The ΔΔG_bind-exp_ values of R225A in the three complexes were much lower than those of ΔΔG_bind-calc_; this binding free energy gap could be attributed to the fact that the corresponding computational calculated electrostatic energy was overestimated. Nevertheless, the ΔΔG_bind-exp_ of R225A in the three complexes were in qualitatively good agreement with those determined by computational site-directed mutagenesis. In contrast, the ΔΔG_bind-calc_ of H129A in the Endothall-PP5c complex was much lower than in other two complexes, its ΔΔG_bind-exp_ was similar with those in other two. Since our experimental result showed that the kcat of H129A mutant decreased by nearly 19-fold supporting the conclusions of recent theoretical studies that His 129 in PP5c and the corresponding His 125 in PP1 are crucial for the catalysis of substrate^25^,^29^, we presumed that the loss of catalytic capability of H129A mutant cause small differences of ΔΔG_bind-exp_ in the three complexes. His 129 in PP5c is vital for cantharidin and its analogs to bind, as well as for catalysis of substrate. In summary, both theoretically and experimentally estimated ΔΔG_bind_ were in qualitative agreement with each other, supporting His 129 and Arg 225 of PP5c as hot-spots that might play important roles in interactions with cantharidin and its analogs.

### Insights for future design of novel inhibitors

As far as we know, studies about human PP5c are extensively conducted^10^,^24^,^25^, while the synthesized novel scaffold inhibitors against PP5c are limited. This study of three inhibitors against PP5c conducted by MD simulations, binding free energy, free energy decomposition, alanine scanning mutagenesis and site-directed mutagenesis bioassays was significant to offer a valuable and deep perspective on the detailed interactions of PP5c with cantharidin-like inhibitors at the atomic level and synthetic novel scaffold inhibitors. Binding free energy analyses suggested that both the electrostatic energy and the van der Waals energy acting as a factor to determine the binding affinities of the ligands to the active pocket of PP5c. The binding mode (Fig. 3 left) and the free energy decomposition of these three complexes showed that the residues His 69, Asn 128, His 129, Arg 225, His 252 and Arg 250 are of importance to inhibit against PP5c.The hydrophobic interactions between the sidechain of Val 254, Phe 271, Tyr 276 came out to be significantly favorable contributions to the total interaction free energies during the whole MD simulation. Interestingly, even though the residue Arg 250 was far away from the active center of PP5c, it contributed a remarkable energy to the total interaction free energy in these three complexes, indicating that the residue Arg 250 could possibly be involved in the formation of the metal center folding for PP5c. These findings indicate that new potent and less cytotoxic anti-cancer cantharidin-like drugs can be designed by enhancing the interactions with these residues as mentioned above. Furthermore, the computational results discussed above demonstrate some important findings: the coordination bonds formed between the manganese ion and three inhibitors are necessary for these to exert the inhibitive interaction; H-bonds established between inhibitors and the residues His 129, Arg 225 and Val 254 play vital roles in stabilizing the complexes; The hydrophobic interactions established between the residues Val 254, Phe 271 and Tyr 276, especially Glu 253 are very important to enhancing the inhibitive interaction;The ASM results obtained were in qualitative agreement with those determined by the experimental site-directed mutagenesis, suggesting that His 129 and Arg 225 are hot-spots that could be exploited to increase the affinity of novel cantharidin-based drugs;The interactions of inhibitor with three negatively charged residues, Asp 99, Glu 130 and Asp 213 around the PP5c active pocket, should be avoided in case to impede their inhibitory activity. However, the interactions of inhibitor with another residue, Arg 250 nearby the active pocket, could enhance the inhibitory activity. Furthermore, it is worth to mention that the two Manganese ions in the coordination center, the MN2 unfavorably contribute to the total interaction free energies. For drug design, the coordination between MN2 and chemical group of inhibitor should be avoided in case of reducing the inhibitory action. Current work provides a better structural understanding of how cantharidin and its analogs bind to PP5c at an atomic level and will contribute to the rational design of new cantharidin-like anti-cancer drugs.

## Methods

The structures for the manganese-containing systems (Figure S2) were taken from the Research Collaboration for Structural Bioinformatics protein database (RCSB Protein Data Bank, PDB ID: 3H63; 3H61; 3H64)^22^, and all the structures were visualized using pymol 1.3 r1 edu. Manganese-related force field parameters were determined using Seminario’s method^30^. Atomic single charge was calculated using the restrained electrostatic potential (RESP) fitting protocol^31^. The Quantum Mechanics (QM) and Molecular Mechanics (MM) vibrational frequencies were compared to check the quality of the force field parameters^30^,^31^,^32^,^33^. We performed three individual 50 ns MD at different seeds for each system. All MD simulations were performed with the AMBER12 package. All MD results were analyzed with the Ambertools13 package based on three individual 50 ns MD trajectories, and the data were averaged. Molecular Mechanics-Poisson-Boltzmann Surface Area (MM-PBSA)^32^ calculations were performed on 1000 snapshots exacted from 40 ~ 50 ns production trajectories with a time interval of 10 ps to estimate the binding free energy. Since only the catalytic domain was used in the simulations, the starting point in our constructed structure corresponds to the 176th residue in the reference structure. To unveil the binding modes of PP5c with cantharidin, norcantharidin and endothall, and to reveal the binding mechanisms at the atomic level, the protein-inhibitor interaction spectrum on a per-residue basis for each inhibitor bound with PP5c was generated by the MM-PBSA binding free energy decomposition analysis. The per-residue energy contribution profiles are further broken down into backbone, side chain, and total interaction free energies to their decomposition energies, which were decomposed per residue into three terms: Van der Waals contribution, electrostatic contribution and polar solvation contribution. The computational alanine scanning mutagenesis (ASM) protocol^33^ was conducted to seek out the key residues involved in inhibitor binding. HuPP5 cDNA was isolated from the liver cancer cell line SMMC-7721 and mutants were generated using the QuikChange® site-directed mutagenesis kit. Wild-type and mutants HuPP5 were expressed in *E.coli* and purified to homogeneity. The IC_50_ values of the three compounds against all HuPP5 proteins were calculated from the dose-dependent inhibition assay. Detailed descriptions of materials and methods are provided in Supplementary Information.

## Additional Information

**How to cite this article**: Liu, J.-Y. *et al.* Insights into the key interactions between human protein phosphatase 5 and cantharidin using molecular dynamics and site-directed mutagenesis bioassays. *Sci. Rep.* **5**, 12359; doi: 10.1038/srep12359 (2015).

## Supplementary Material

Supplementary Information

## Figures and Tables

**Table 1:** Hydrogen bonds formed between inhibitors (Cantharidin, Norcantharidin and Endothall) and PP5c during MD simulations^a^.

**System**	**DONOR**	**ACCEPTORH**	**ACCEPTOR**	**Occupancy(%)**	**Distance (Å)**	**Angle (°)**
	**res@atom**	**res@atom**	**res@atom**			
**Cantharidin-PP5c**						
	318@O5	225@HH11	225@NH1	97.09	2.82 (0.12)	33.19 (11.71)
	318@O4	129@HE2	129@NE2	60.03	2.77 (0.11)	40.23 (13.50)
**Norcantharidin-PP5c**						
	318@O5	225@HH11	225@NH1	93.41	2.77 (0.11)	31.60 (12.35)
	318@O3	129@HE2	129@NE2	70.47	2.77 (0.12)	41.20 (11.81)
	318@O4	129@HE2	129@NE2	19.05	3.13 (0.19)	53.5 (5.38)
**Endothall-PP5c**						
	318@O5	254@H	254@N	69.84	2.92 (0.15)	41.01 (11.88)
	318@O3	225@HH11	225@NH1	63.22	2.87 (0.20)	26.45 (10.24)

^a^The percentage of simulation snapshots (saved every 10 ps) in which the H-bond was present are listed.

Hydrogen bonds are determined by an acceptor/donor atom distance of less than 3.5 Å and an acceptor/H–donor angle of greater than 120°. The occupancy of H-bonds that are formed between the inhibitors and the PP5c larger than 5% is listed.

**Table 2:** The theoretical and experimental ΔΔG_bind_^a^ value for WT and mutant complexes.

**Protein**	**R100A**	**H129A**	**R225A**	**R250A**	**V254A**	**D99A**	**E130A**	**D213A**
**Cantharidin-PP5c**								
ΔΔG_bind-calc_	1.05	14.68	10.38	2.06	–	−1.84	−3.76	−2.38
ΔΔG_bind-exp_^b^	0.97	>5.74	2.03	–	–	–	–	–
**Norcantharidin-PP5c**								
ΔΔG_bind-calc_	1.02	11.36	10.45	2.29	–	−2.27	−4.65	−3.14
ΔΔG_bind-exp_^b^	0.88	>4.75	2.24	–	–	–	–	–
**Endothall-PP5c**								
ΔΔG_bind-calc_	1.31	3.42	12.26	2.68	1.82	−1.13	−2.53	−2.48
ΔΔG_bind-exp_^b^	0.99	>4.83	3.46	–	–	–	–	–

^a^All values are given in kcal/mol.

^b^The binding free energy difference (ΔΔG_bind_) between the mutant and wild type complexes is defined as ΔΔG_bind_ = RTln(IC_50_ mutant/IC_50_ wild-type), where R is the ideal gas constant and T is the temperature in K.
