# Supplementary material for: Insights into the key interactions between human protein phosphatase 5 and cantharidin using molecular dynamics and site-directed mutagenesis bioassays
Source: Sci Rep. 2015 Jul 20;5:12359. doi: 10.1038/srep12359 (PMC4507179; doi:10.1038/srep12359)
Supplement: Supplementary Information [file srep12359-s1.pdf]

Insights into the key interactions between human protein phosphatase 5 and cantharidin using molecular dynamics and site-directed mutagenesis bioassays

Ji-Yuan Liu[1a], *Xi-En Chen* [1a], *Ya-Lin Zhang*[1*]

[1] Key Laboratory of Plant Protection Resources & Pest Management of the Ministry of Education, Northwest A&F University, Yangling 712100, Shaanxi, China;

[a] These authors contributed equally to this work.

Ya-Lin Zhang, E-mail: yalinzh@nwsuaf.edu.cn.

*Corresponding Author

Key Laboratory of Plant Protection Resources & Pest Management of the Ministry of Education, Northwest A&F University, Yangling 712100, Shaanxi, China. Tel./Fax: +86-29-8709-2190. E-mail: yalinzh@nwsuaf.edu.cn.

Disclosure of Potential Conflicts of Interest

No potential conflicts of interest were disclosed.

Supplementary Tables

Table S1. Bond-stretching and angle-bending parameters related to manganese derived from the chosen models.

**Bond type** [a]	**Stretching force constant kcal/(mol· Å^2)**	**Equilibrium bond length (Å)**
**Model I-Cantharidin**		
MN-NB	41.64	2.234
MN-O2	50.25	2.130
MN-O	24.53	2.189
MN-o	28.92	2.221
MN-os	20.17	2.246
**Model I-Norcantharidin**		
MN-NB	47.53	2.229
MN-O2	34.26	2.187
MN-O	25.85	2.182
MN-o	29.28	2.206
MN-os	19.53	2.315
**Model I-Endothall**		
MN-NB	49.49	2.203
MN-O2	29.01	2.218
MN-O	21.58	2.190
MN-o	21.04	2.869
MN-os	13.53	2.340
**Bond angle** [a]	**Bending force constant kcal/(mol· rad^2)**	**Equilibrium bond angle (°)**
**Model I-Cantharidin**		
C -O -MN	38.557	127.945
NB-MN-O	28.083	125.226
NB-MN-o	29.051	111.332
NB-MN-NB	22.935	97.254
CV-NB-MN	45.986	125.750
CR-NB-MN	42.114	119.433
o -MN-o	43.215	80.763
o -MN-os	35.526	79.851
MN-O2-MN	27.664	107.162
MN-o -MN	54.054	100.442
MN-o -c	72.335	125.560
CC-NB-MN	40.710	133.097
O2-MN-o	37.235	105.000
O2-MN-os	23.797	121.546
O2-MN-NB	26.450	110.723

O2-MN-O	25.756	85.480
O2-MN-O2	25.751	94.344
NB-MN-os	23.977	109.720
MN-os-c3	44.687	119.208
O -MN-o	48.859	104.769
C -O2-MN	72.041	129.982
**Model I-Norcantharidin**		
C -O -MN	37.264	135.224
NB-MN-O	26.785	126.590
NB-MN-o	33.923	113.376
NB-MN-NB	27.299	95.143
CV-NB-MN	43.620	129.023
os-MN-o	31.480	83.261
CR-NB-MN	43.319	120.114
MN-O2-MN	24.780	101.181
MN-o -MN	57.927	102.650
MN-o -c	78.450	123.346
CC-NB-MN	46.665	134.108
O2-MN-o	35.996	103.383
O2-MN-os	24.802	122.328
O2-MN-NB	28.152	113.160
O2-MN-O	32.837	86.199
O2-MN-O2	21.510	98.270
o -MN-o	36.208	80.539
NB-MN-os	28.340	93.442
MN-os-c3	46.472	115.560
O -MN-o	49.254	102.422
C -O2-MN	57.147	132.536
**Model I-Endothall**		
C -O -MN	59.494	134.319
NB-MN-O	21.128	93.649
NB-MN-o	29.546	119.960
NB-MN-NB	22.115	97.863
CV-NB-MN	42.585	126.627
os-MN-o	31.251	81.820
CR-NB-MN	44.669	120.960
MN-O2-MN	55.527	86.904
MN-o -MN	44.387	43.373
MN-o -c	72.214	118.462
CC-NB-MN	48.547	129.604
O2-MN-o	35.743	95.677
O2-MN-os	31.054	135.957
O2-MN-NB	30.804	119.087
O2-MN-O	21.234	91.459

O2-MN-O2	33.559	74.710
o -MN-o	33.401	86.957
NB-MN-os	24.204	87.545
MN-os-c3	47.557	114.992
O -MN-o	34.261	132.958
C -O2-MN	87.818	130.688

[a] The atom types used in this table are indicated in Figure S3

Table S2. Calculated average distances between manganese and its ligated atoms of three complexes (in Å) compared with X-ray experimental values [a]

MN1	MD	X-Ray	MN2	MD	X-Ray
**Cantharidin-PP5c**					
Cantharidin@O3	2.16±0.08	2.0	Cantharidin@O1	2.20±0.07	2.2
Asp 96@OD2	2.16±0.05	2.3	Cantharidin@O2	2.16±0.07	2.1
Asn 128@OD1	2.11±0.08	2.1	Cantharidin@O3	2.35±0.05	2.1
His 177@NE2	2.37±0.05	2.1	Asp 67@OD2	2.02±0.05	2.1
His 252@ND1	2.30±0.06	2.2	His 69@NE2	2.23±0.05	2.2
			Asp 96@OD2	2.15±0.05	2.2
**Norcantharidin-PP5c**					
Norcantharidin@O2	2.07±0.06	2.0	Norcantharidin@O1	2.27±0.07	2.3
Asp 96@OD2	2.22±0.06	2.3	Norcantharidin@O2	2.22±0.05	2.2
Asn 128@OD1	2.15±0.08	2.0	Norcantharidin@O4	2.04±0.07	2.2
His 177@NE2	2.35±0.05	2.1	Asp 67@OD2	1.99±0.07	2.1
His 252@ND1	2.31±0.05	2.2	His 69@NE2	2.24±0.05	2.2
			Asp 96@OD2	2.13±0.06	2.2
**Endothall-PP5c**					
Endothall@O4	2.63±0.07	2.3	Endothall@O1	2.38±0.09	2.6
Asp 96@OD2	1.96±0.05	2.4	Endothall@O2	2.87±0.07	2.4
Asn 128@OD1	2.23±0.07	2.0	Endothall@O4	2.48±0.08	2.4
His 177@NE2	2.27±0.05	2.1	Asp 67@OD2	2.02±0.06	2.0
His 252@ND1	2.28±0.05	2.2	His 69@NE2	2.22±0.05	2.1
			Asp 96@OD2	2.17±0.06	2.2

[a] The labels of residues and the names of the atoms adopted from PDB convention are given in Figure S4.

Table S3. The estimated binding free energy for Cantharidin-PP5c, Norcantharidin-PP5c and Endothall-PP5c complexes.

Contribution[a]	Cantharidin-PP5c	Norcantharidin-PP5c	Endothall-PP5c
ΔE_{vdW}	5.06 (1.03)	29.38 (0.98)	-1.31 (0.78)
ΔE_{ele}	-124.42 (0.91)	-209.03 (0.88)	-131.7 (0.81)
ΔG_{EPB}	104.92 (0.74)	174.33 (0.62)	120.96 (0.48)
ΔG_{CAVITY}	-2.23 (0.0014)	-2.05 (0.0015)	-1.87 (0.0023)
ΔG_{gas}	-119.36 (1.19)	-179.64 (0.99)	-133.00 (1.07)
ΔG_{sol}	102.70 (0.75)	172.28 (0.72)	119.09 (0.52)
$\Delta G_{bind\text{-}calc}$	-16.67 (0.91)	-7.36 (0.89)	-13.91 (0.83)

[a] All energies are in kcal/mol with standard errors in parentheses.

Table S4. Decomposition of binding free energy on a per-residue level [a]

Residue	S_{VDW}	B_{VDW}	T_{VDW}	S_{ELE}	B_{ELE}	T_{ELE}	S_{EPB}	B_{EPB}	T_{EPB}	S_{TOT}	B_{TOT}	T_{TOT}
**Cantharidin-PP5c**												
**Asp 67**	-1.02	0	-1.02	25.33	0	25.33	-21.37	0	-21.37	2.94	0	2.94
**His 69**	-1.44	0	-1.44	-9.96	0	-9.96	8.82	0	8.82	-2.58	0	-2.58
**Asp 96**	-0.48	0	-0.48	22.12	0	22.12	-17.5	0	-17.5	4.13	0	4.13
**Asp 99**	-0.04	-0.034	-0.074	17.77	3.83	21.60	-16.92	-3.61	-20.53	0.81	0.18	1.00
**Arg 100**	-0.091	-0.024	-0.12	-16.79	0.47	-16.32	16.28	-0.46	15.82	-0.61	-0.011	-0.62
**Asn 128**	-1.17	0	-1.17	-11.21	0	-11.21	10.67	0	10.67	-1.70	0	-1.70
**His 129**	0.02	-0.075	-0.056	-41.07	-2.61	-43.68	36.77	2.18	38.95	-4.28	-0.51	-4.79
**Glu 130**	-0.019	-0.01	-0.03	20.90	1.75	22.65	-18.93	-1.68	-20.62	1.95	0.05	2.00
**His 177**	-0.35	0	-0.35	10.94	0	10.94	-8.44	0	-8.44	2.16	0	2.16
**Asp 213**	-0.049	-0.009	-0.057	19.82	2.14	21.96	-18.45	-2.01	-20.47	1.32	0.12	1.43
**Arg 225**	-0.19	-0.015	-0.20	-37.28	0.99	-36.29	32.91	-0.96	31.96	-4.56	0.019	-4.54
**Arg 250**	-0.041	-0.008	-0.048	-19.74	-0.23	-19.96	18.37	0.10	18.48	-1.41	-0.13	-1.54
**His 252**	-2.13	0	-2.13	-14.14	0	-14.14	13.14	0	13.14	-3.12	0	-3.12
**Glu 253**	-0.25	-0.95	-1.20	15.96	3.52	19.47	-15.43	-2.45	-17.88	0.28	0.13	0.40
**Val 254**	-0.45	-0.40	-0.84	-2.18	2.07	-0.11	2.22	-1.94	0.29	-0.40	-0.26	-0.66
**Phe 271**	-0.82	-0.025	-0.85	-1.08	-0.57	-1.65	1.11	0.52	1.63	-0.80	-0.08	-0.88
**Tyr 276**	-1.57	-0.041	-1.61	-1.59	0.28	-1.31	3.04	-0.23	2.81	-0.12	-0.01	-0.11
**MN1**	5.17	0	5.17	-50.87	0	-50.87	39.02	0	39.02	-6.68	0	-6.68
**MN2**	11.43	0	11.43	-54.36	0	-54.36	45.21	0	45.21	2.28	0	2.28

**Norcantharidin-PP5c**												
**Asp 67**	-1.1	0	-1.1	32.27	0	32.27	-26.93	0	-26.93	4.24	0	4.24
**His 69**	-1.36	0	-1.36	-10.96	0	-10.96	9.68	0	9.68	-2.64	0	-2.64
**Asp 96**	0.22	0	0.22	30.66	0	30.66	-23.93	0	-23.93	6.94	0	6.94
**Asp 99**	-0.036	-0.033	-0.068	20.43	4.67	25.10	-19.51	-4.38	-23.89	0.88	0.26	1.14
**Arg 100**	-0.17	-0.021	-0.19	-24.98	0.63	-24.35	24.86	-0.63	24.22	-0.29	-0.021	-0.31
**Asn 128**	-0.96	0	-0.96	-3.67	0	-3.67	4.39	0	4.39	-0.23	0	-0.23
**His 129**	0.126	-0.06	0.067	-48.03	-2.49	-50.51	42.74	2.10	44.84	-5.16	-0.45	-5.61
**Glu 130**	-0.017	-0.09	-0.025	23.49	2.19	25.68	-21.21	-2.10	-23.32	2.26	0.074	2.34
**His 177**	-0.38	0	-0.38	4.13	0	4.13	-3.10	0	-3.10	0.65	0	0.65
**Asp 213**	-0.034	-0.007	-0.041	21.78	2.30	24.08	-20.3	-2.18	-22.47	1.45	0.12	1.57
**Arg 225**	0.32	-0.01	0.31	-42.33	1.33	-41.01	36.58	-1.27	35.31	-5.43	0.042	-5.39
**Arg 250**	-0.041	-0.008	-0.049	-22.47	-0.27	-22.37	20.98	0.15	21.13	-1.53	-0.13	-1.66
**His 252**	-1.82	0	-1.82	-14.81	0	-14.81	14.10	0	14.10	-2.53	0	-2.53
**Glu 253**	-0.20	-0.88	-1.08	18.11	3.80	21.92	-17.5	-2.92	-20.42	0.42	0.0035	0.42
**Val 254**	-0.45	-0.29	-0.74	-2.45	2.30	-0.16	2.47	-2.20	0.26	-0.44	-0.19	-0.63
**Phe 271**	-0.77	-0.027	-0.79	-1.24	-0.65	-1.89	1.17	0.57	1.73	-0.84	-0.11	-0.95
**Tyr 276**	-1.42	-0.039	-1.46	-1.83	-0.42	-2.25	3.92	0.41	4.33	0.67	-0.049	0.62
**MN1**	10.54	0	10.54	-70.25	0	-70.25	54.41	0	54.41	-5.30	0	-5.30
**MN2**	18.17	0	18.17	-78.3	0	-78.3	61.84	0	61.84	1.70	0	1.70
**Endothall-PP5c**												
**Asp 67**	-1.22	0	-1.22	28.3	0	28.3	-24.04	0	-24.04	3.04	0	3.04

**His 69**	7.46	0	7.46	-8.66	0	-8.66	8.64	0	8.64	7.44	0	7.44
**Asp 96**	-0.84	0	-0.84	27.55	0	27.55	-26.8	0	-26.8	-0.082	0	-0.082
**Asp 99**	-0.032	-0.046	-0.077	15.86	4.23	20.10	-15.41	-3.92	-19.32	0.43	0.27	0.70
**Arg 100**	-0.17	-0.035	-0.20	-17.85	1.06	-16.79	17.21	-0.92	16.30	-0.80	0.103	-0.70
**Asn 128**	-0.38	0	-0.38	-8.48	0	-8.48	7.78	0	7.78	-1.08	0	-1.08
**His 129**	-0.63	-0.036	-0.66	-30.15	-1.90	-32.05	30.50	1.83	32.33	-0.27	-0.112	-0.39
**Glu 130**	-0.013	-0.007	-0.019	19.61	1.95	21.56	-18.85	-1.92	-20.77	0.74	0.030	0.77
**His 177**	-0.22	0	-0.22	4.72	0	4.72	-4.03	0	-4.03	0.47	0	0.47
**Asp 213**	-0.013	-0.004	-0.017	18.98	2.09	21.07	-18.12	-2.01	-20.13	0.84	0.078	0.92
**Arg 225**	-0.52	-0.011	-0.534	-40.51	0.84	-39.67	36.89	-0.83	36.07	-4.14	0.004	-4.14
**Arg 250**	-0.027	-0.007	-0.033	-24.21	-0.15	-24.36	22.55	0.07	22.62	-1.69	-0.091	-1.78
**His 252**	-1.95	0	-1.95	-21.61	0	-21.61	18.42	0	18.42	-5.13	0	-5.13
**Glu 253**	-0.26	-0.51	-0.76	26.60	1.68	28.27	-25.11	-2.08	-27.2	1.22	-0.91	0.31
**Val 254**	-0.70	0.07	-0.77	-3.90	1.87	-2.02	3.34	-2.37	0.97	-1.25	-0.57	-1.82
**Phe 271**	-0.70	-0.026	-0.73	-1.32	-0.73	-2.06	1.20	0.55	1.75	-0.82	-0.21	-1.03
**Tyr 276**	-0.95	-0.041	-0.99	-0.52	-0.17	-0.69	1.78	0.096	1.88	0.31	-0.12	0.20
**MN1**	0.23	0	0.23	-50.6	0	-50.6	46.48	0	46.48	-3.89	0	-3.89
**MN2**	2.39	0	2.39	-56.99	0	-56.99	54.99	0	54.99	0.39	0	0.39

[a] Energies shown as contributions from van der Waals energy (VDW), electrostatic energy (ele), polar solvation energy (EPB) and total energy (TOT) of sidechain atoms (S), backbone atoms (B), and the sum of them (T) for Cantharidin-PP5c, Norcantharidin-PP5c, and Endothall-PP5c complexes. All values are given in kcal/mol.

Table S5. Primers used in PCR-based site-directed mutagenesis

Primer name	Sequence(5'--3')
R100A-F	GGTGACTTTGTGGACgcaGGCTCCTTCTCTGTAG
R100A-R	CTACAGAGAAGGAGCCtgcGTCCACAAAGTCACC
H129A-F	CCTCCTTCGAGGCAACgccGAGACAGACAACATG
H129A-R	CATGTTGTCTGTCTCggcGTTGCCTCGAAGGAGG
R225A-F	GCGCTCGATCAGCAAGgcgGGCGTGAGCTGTCAG
R225A-R	CTGACAGCTCACGCCcgcCTTGCTGATCGAGCGC

Mutation sites are indicated by lowercase.

Table S6. Enzyme kinetic properties of recombinant wild HuPP5 and mutations.

	*K*m (mM)	*V*max (μmol/min/mg)	*k*cat (S^{-1})	*k*cat/*K*m ($M^{-1}S^{-1}$)
Wild	5.84±0.12	6.77±0.26	6.42	1099.32
R100A	27.53±1.37	1.28±0.03	1.21	43.95
H129A	27.64±2.27	0.36±0.04	0.34	12.3
R225A	10.25±0.34	1.43±0.13	0.95	93.68

All results are presented as the mean ± S.D. for three independent experiments.

Table S7. Inhibition (IC_{50}) of recombinant wild-type HuPP5 and mutants.

Compound	IC50 (μM)			
	Wild	R100A	H129A	R225A
Cantharidin	0.82±0.06	4.05±0.34	>10 mM	21.92±1.12
Norcantharidin	4.18±0.25	17.76±1.44	>10 mM	164.5±9.84
Endothall	3.64±0.17	18.35±0.39	>10 mM	1064.2±63.72

All results are presented as the mean ± S.D. for three independent experiments.

Supplementary Figures

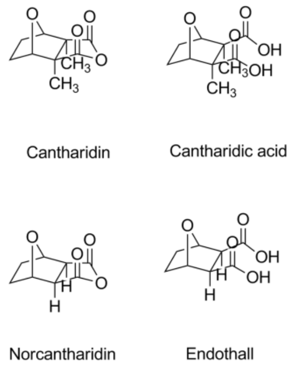

Figure S1. Structures of cantharidin, norcantharidin together with their open dicarboxylic acid forms, cantharidic acid and endothall, respectively.

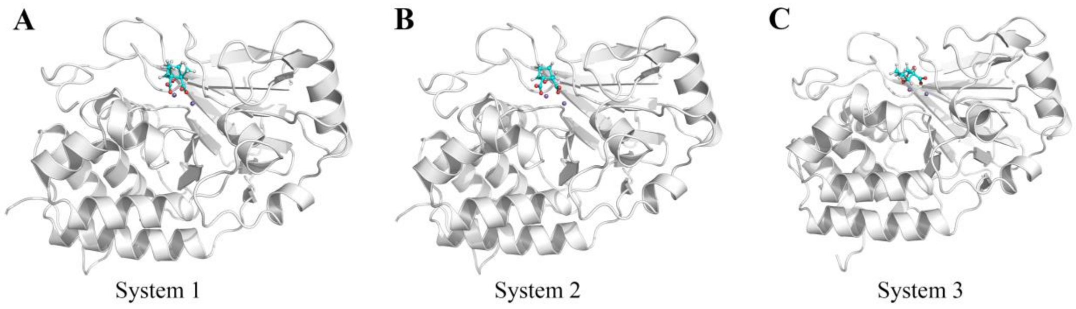

Figure S2. Ribbon representation of PP5c complexes with cantharidin (A), norcantharidin (B) and endothall (C). The manganese ions are presented as slate spheres. Cantharidin, norcantharidin and endothall are presented with the stick model. Color code: cyan, C; red, O; white, H.

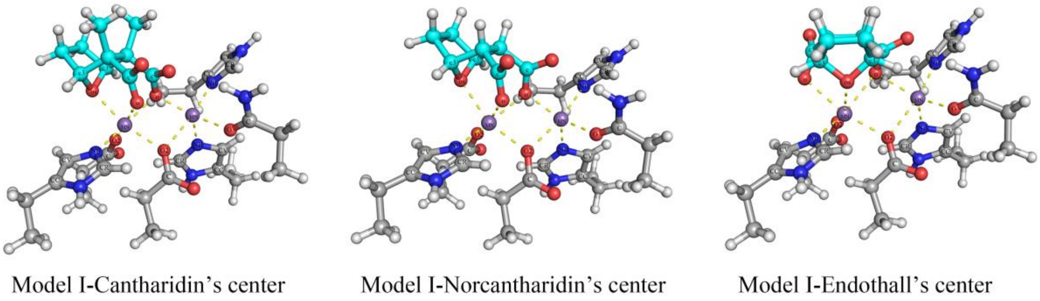

Figure S3. Three models used for derivation of manganese-related force field parameters. Atom types are labeled in lower cases. The manganese ions are presented as slate spheres. Cantharidin, norcantharidin and endothall are presented with the stick-and-sphere model. Color code: cyan, C; red, O; white, H. The residues which ligated the manganese ions are also presented with the stick-and-sphere model. Color code: gray, C; red, O; blue, N; white, H; yellow dashed line, coordination bond.

Model II-Cantharidin's center

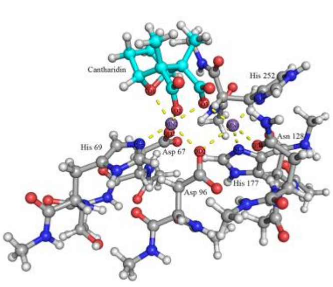

Residue	Charge
Asp 67	-0.40
His 69	0.26
Asp 96	-0.63
Asn 128	0.22
His 177	-0.14
His 252	0.44
MN1	0.70
MN2	0.70
Cantharidin	-1.16

Model II-Norcantharidin's center

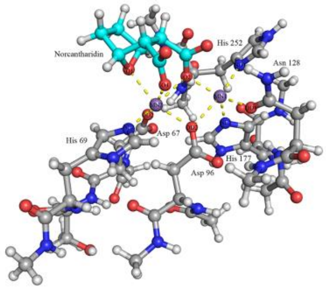

Residue	Charge
Asp 67	-0.50
His 69	0.27
Asp 96	-0.66
Asn 128	0.13
His 177	0.0055
His 252	0.40
MN1	0.83
MN2	0.83
Norcantharidin	-1.32

Model II-Endothall's center

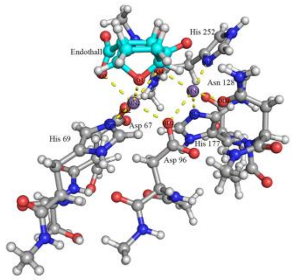

Residue	Charge
Asp 67	-0.56
His 69	0.30
Asp 96	-0.55
Asn 128	0.11
His 177	-0.0044
His 252	0.43
MN1	0.77
MN2	0.77
Endothall	-1.27

Figure S4. Representation of the single point Merz−Kollman charges fitted with the RESP methodology for three models; global charge of the residues for the three models are assigned in atomic units (a.u.). The manganese ions are presented as slate spheres. Cantharidin, norcantharidin and endothall are presented with the stick-and-sphere model. Color code: cyan, C; red, O; white, H. The residues which ligated the manganese ions are also presented with the stick-and-sphere model. Color code: gray, C; red, O; blue, N; white, H; yellow dashed line, coordination bond.

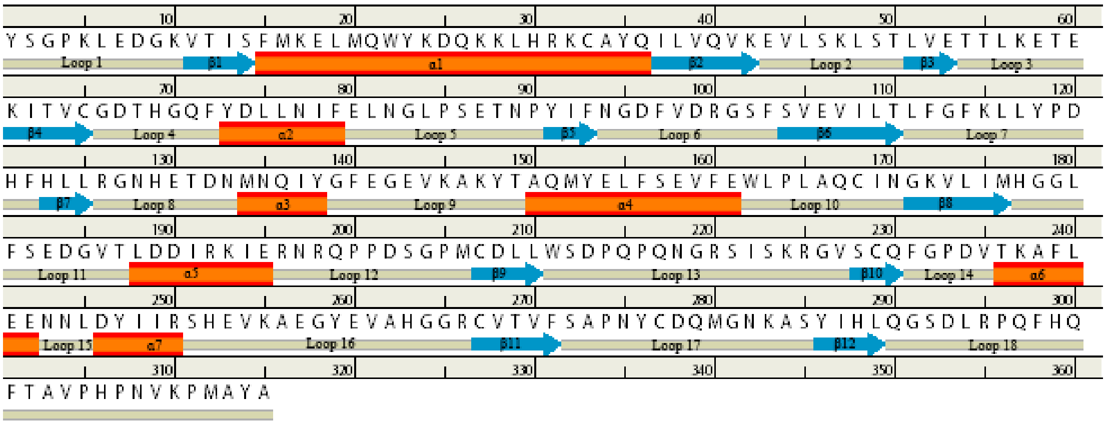

Figure S5. The secondary structure of PP5c. α helixes and β sheets are shown in red boxes and blue arrows.

Cantharidin-PP5c

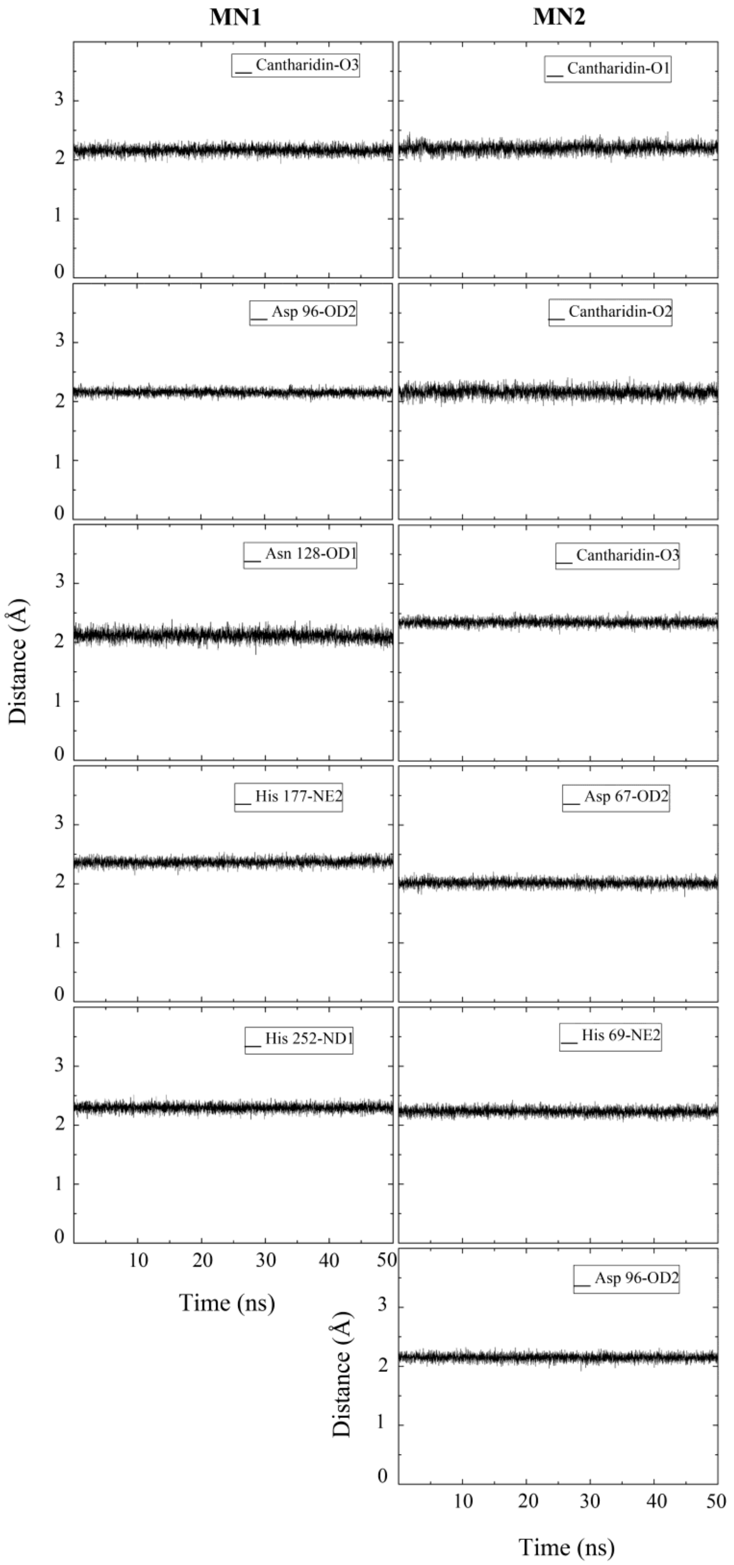

Norcantharidin-PP5c

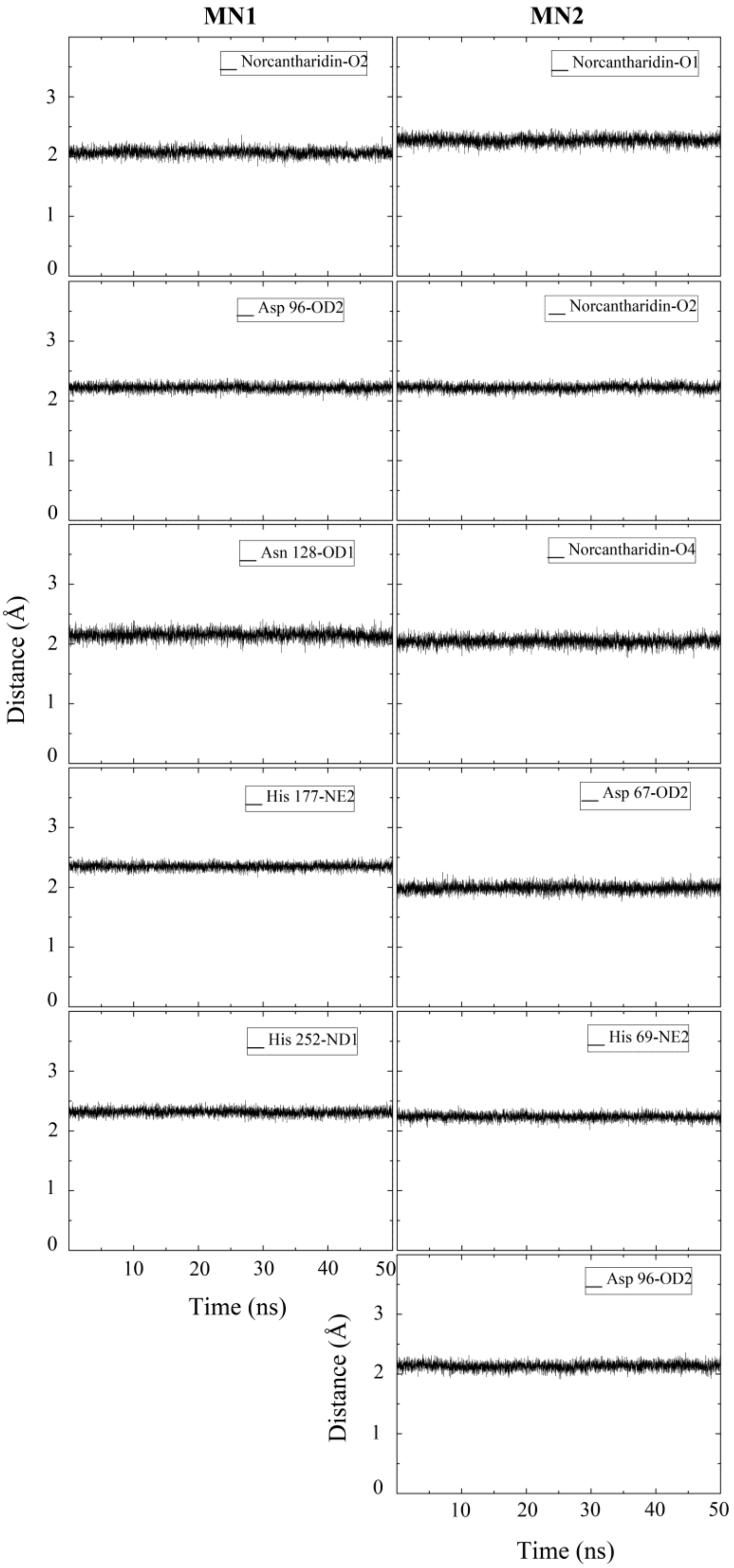

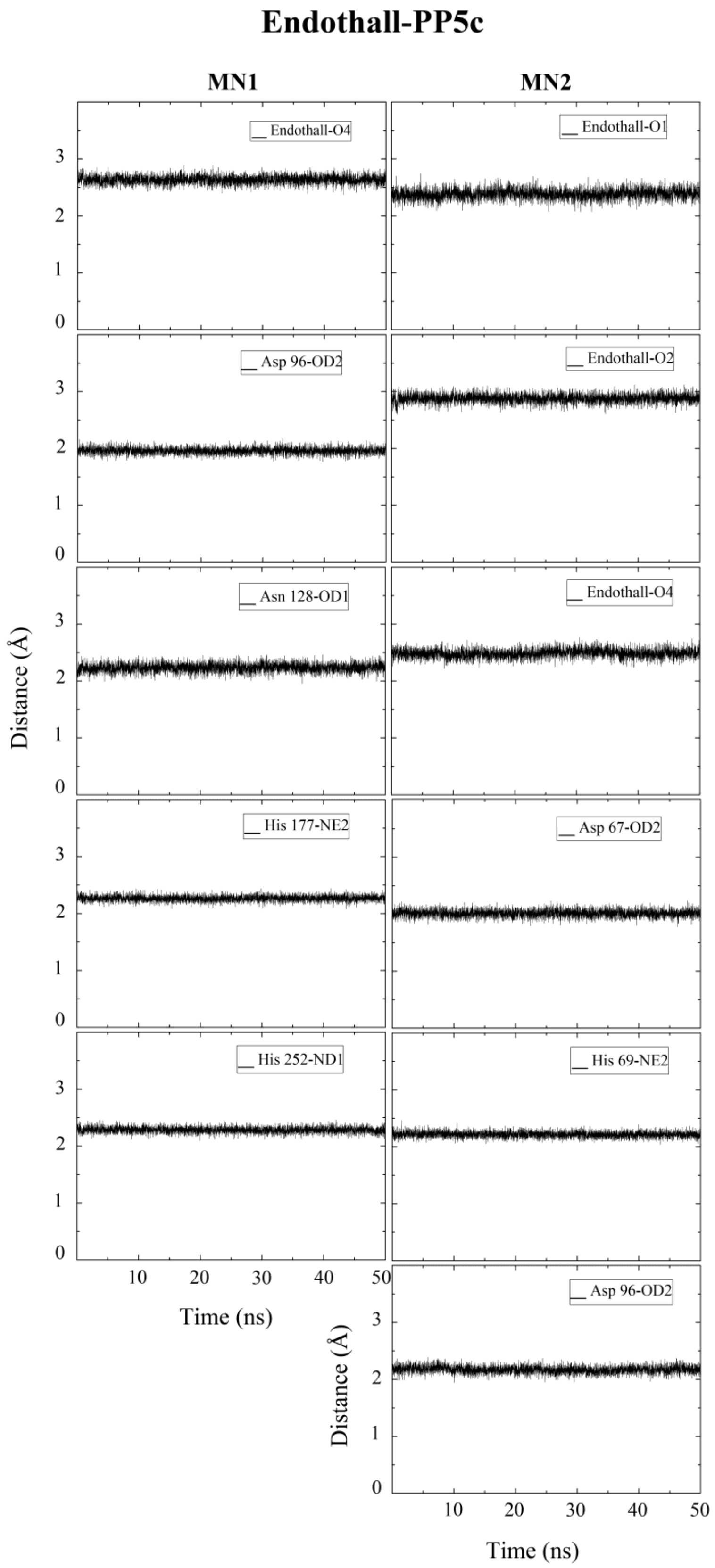

Figure S6. Time-dependence of the coordination bond lengths of the three complexes under the 50 ns MD simulations.

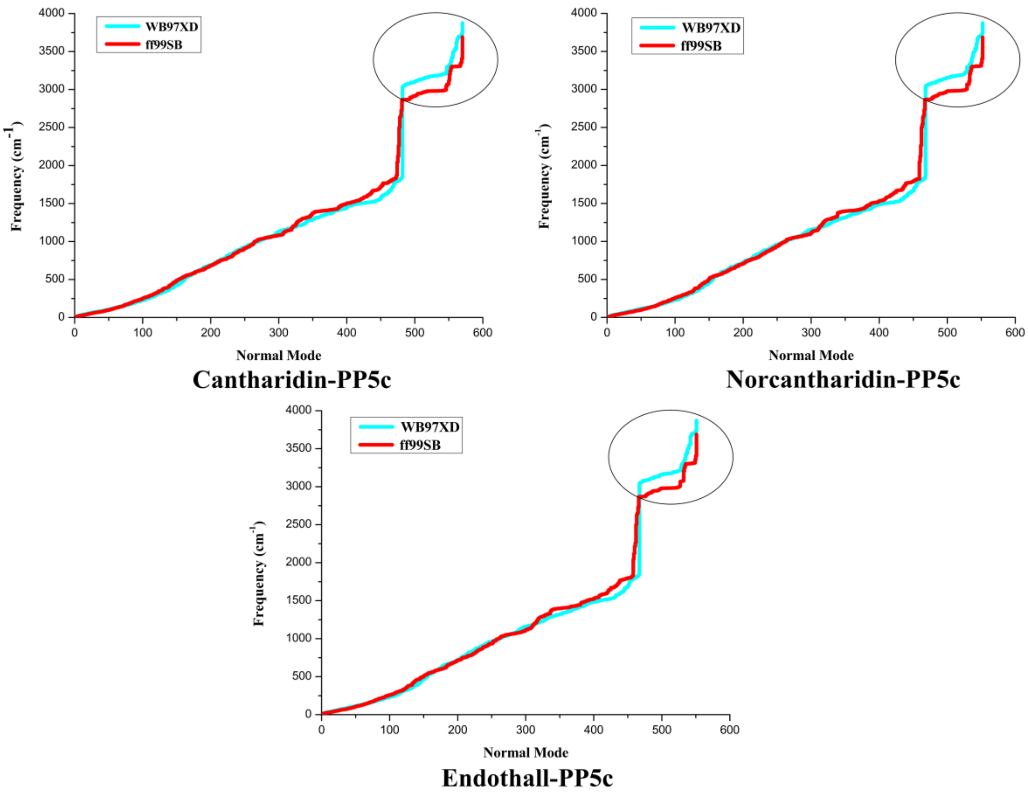

Figure S7. Comparison of the vibrational frequencies of the three model II systems computed at the QM level (WB97XD/6-31G**, in blue) with MM (ff99SB, in red).

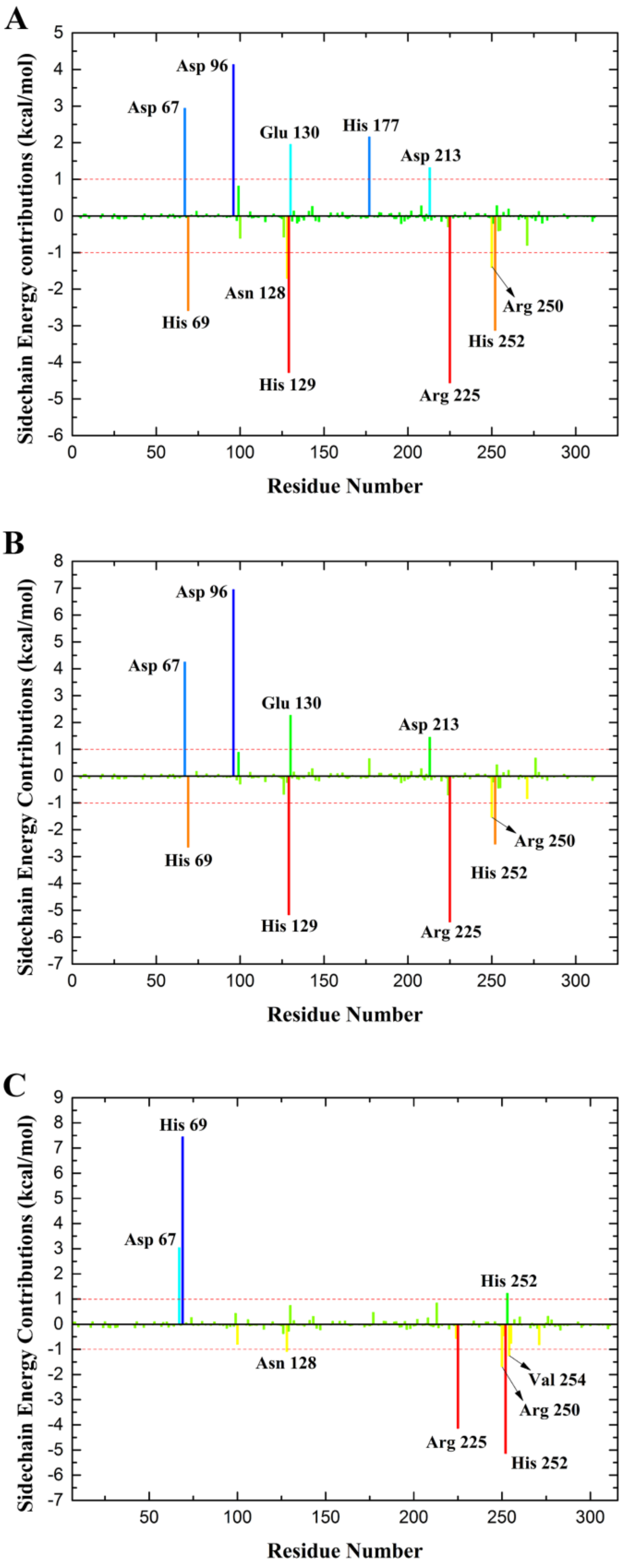

Figure S8. Residue-ligand interaction spectrum of (A) the Cantharidin-PP5c complex, (B) the Norcantharidin-PP5c complex and (C) the Endothall-PP5c complex according to the MM-PBSA method. The x-axis denotes the residue number of the PP5c and the y-axis denotes the sidechain energy contribution for each residue.

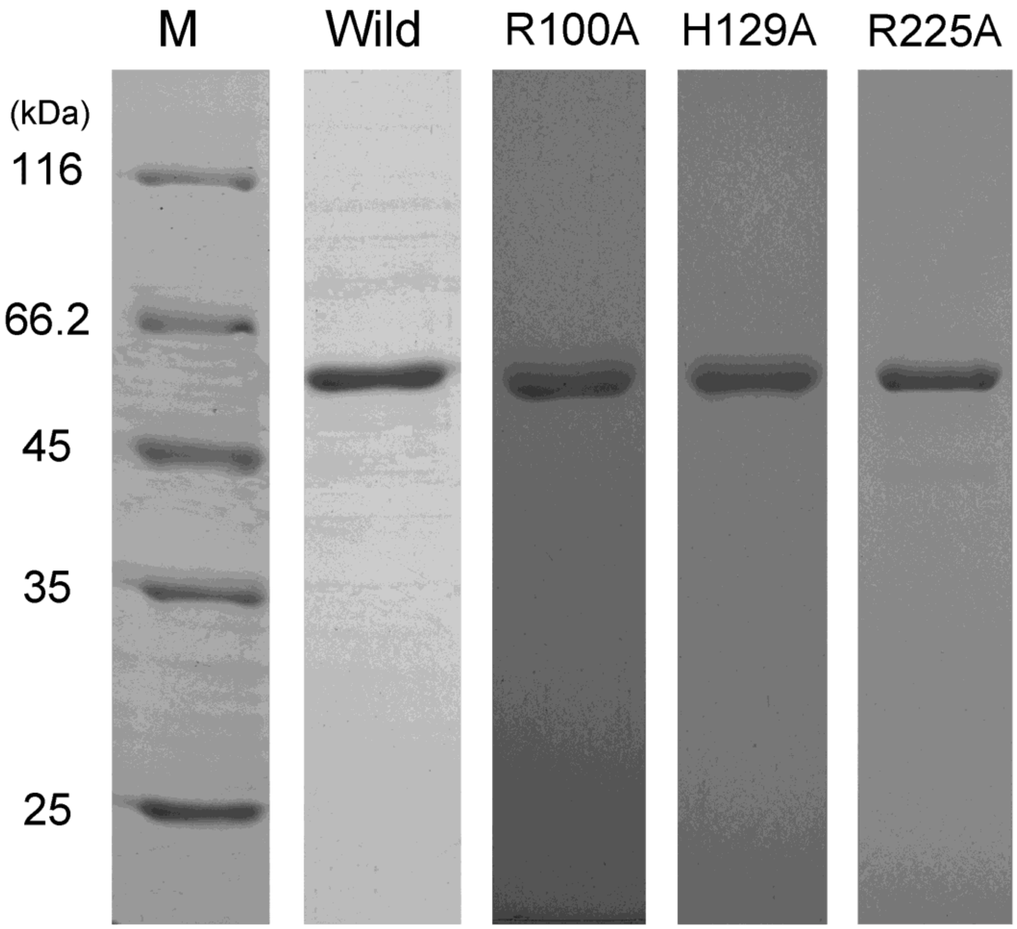

Figure S9. Purification of recombinant wild-type HuPP5 and three mutants.

Supplementary Methods and Materials

Selection of manganese-containing systems. The structures for the manganese-containing systems were taken from the Research Collaboration for Structural Bioinformatics protein database (RCSB Protein Data Bank). The X-ray structure of the catalytic domain of human Serine/Threonine Phosphatase 5 (PP5c) was soaked with four ligands, cantharidin, norcantharidin together with their open dicarboxylic acid forms, cantharidic acid and endothall (Figure S1). Due to cantharidin in its open form and cantharidin acid sitting in the catalytic site of PP5c sharing a very similar pose, we only chose the structure of PP5c with two Mn^{2+} atoms originally soaked with cantharidin as the system 1 (PDB ID 3H63, resolution of 1.3 Å) (Figure S2-A). The norcantharidin had a single pose since it lacked the two methyl groups in contrast to cantharidin, the chosen system 2 (Figure S2-B) was the structure of PP5c with two Mn^{2+} atoms originally soaked with norcantharidin (PDB ID 3H61, resolution of 1.4 Å). The structure of PP5c with two Mn^{2+} atoms originally soaked with endothall was chosen as the system 3 (PDB ID 3H64, resolution of 1.9 Å) (Figure S2-C). Endothall appears to be rotated by 120° with respect to the hydrolyzed forms of cantharidin, cantharidic acid, and norcantharidin.[1]

Derivation of manganese-related force field parameters. The Mn (II)-Mn (II) center that derived from our three systems was bound with three His residues, two Asp residues, and one Asn residue on the protein side. For convenience, the residues bound to the manganese ion were approximated and we used three ethyl imidazole molecules to mimic the side chains of the three His residues. Two propionic acid molecules were used to mimic the side chains of two Asp residues, and a propionamide molecule was used to mimic the side chain of the Asn residue. On the ligand side, the integral structures of three small molecules (Cantharidin, Norcantharidin, and

Endothall) were retained in our three systems. Cantharidin and Norcantharidin were present in the hydrolyzed form. We established three models (Model I) summarized in Figure S3 to perform QM computations. All QM computations were performed using the Gaussian 09 program package.[2] The center of PP5c contains two metal Mn (II), five electrons occupied the 3d orbitals of each Mn (II).Due to the total energy for the antiferromagnetic coupling being higher than for the ferromagnetic coupling by calculating the stable geometries of energy minimization, only the ferromagnetic coupling was considered for the Mn (II)-Mn (II) center. The parallel spins on each Mn^{2+} were ferromagnetically coupled.[3] The charge of cantharidin, norcantharidin, and endothall is -2 in three models. The total charge in the three models is 0, and the total spin S is 11/2 for the ferromagnetically coupled state. Three models were optimized with the long-range corrected hybrid density-functional theory (DFT) method WB97XD.[4] The basic set 6-31G** was used for the carbon (C), hydrogen (H), oxygen (O), and nitrogen atoms (N). The basic set LANL2DZ was employed for manganese ions (Mn). After the geometry optimization, frequency analysis was performed at the same level to confirm the existence of true local minima without any imaginary frequency. The frequency analysis also produced the Cartesian Hessian matrices that were required for the following step.

The bond and angle force constants (K_r and K_θ) were obtained using Seminario's method. K_r and K_θ are derived from the Cartesian Hessian matrices that obtained from the frequency analysis. The derivations of manganese-related force field parameters are compatible with the AMBER force field. The energy function is given by equation (1)

$$E=\sum_{bonds} K_r(r-r_{eq})^2+\sum_{angles} K_\theta(\theta-\theta_{eq})^2+\sum_{dihedrals}\frac{V_n}{2}[1+\cos(\mathrm{n}\phi-\gamma)]+\sum_{i<j}^{atoms}\left[\frac{A_{ij}}{R_{ij}^{12}}-\frac{B_{ij}}{R_{ij}^{6}}+\frac{q_i q_j}{\varepsilon R_{ij}}\right]$$

(1)

The four terms in equation (1) relate to the energies of bond stretching, angle bending, dihedrals and nonbonded van der Waals and electrostatic interactions, respectively.[5] In our study, we derived bond-stretching and angle-bending parameters for manganese ion. According to the Seminario's method, the bond-stretching force constants of bond A-B can be extracted the 3×3 submatrix from the Hessian matrix in Cartesian coordinates.

$$\begin{bmatrix} \delta F_{\mathrm{Ax}} \\ \delta F_{\mathrm{Ay}} \\ \delta F_{\mathrm{Az}} \end{bmatrix} = - \begin{bmatrix} \frac{\partial^2 E}{\partial x_{\mathrm{A}} \partial x_B} & \frac{\partial^2 E}{\partial x_{\mathrm{A}} \partial y_B} & \frac{\partial^2 E}{\partial x_{\mathrm{A}} \partial z_B} \\ \frac{\partial^2 E}{\partial y_{\mathrm{A}} \partial x_B} & \frac{\partial^2 E}{\partial y_{\mathrm{A}} \partial y_B} & \frac{\partial^2 E}{\partial y_{\mathrm{A}} \partial z_B} \\ \frac{\partial^2 E}{\partial z_{\mathrm{A}} \partial x_B} & \frac{\partial^2 E}{\partial z_{\mathrm{A}} \partial y_B} & \frac{\partial^2 E}{\partial z_{\mathrm{A}} \partial z_B} \end{bmatrix} \times \begin{bmatrix} \delta x_B \\ \delta y_B \\ \delta z_B \end{bmatrix} \quad (2)$$

Or in compact notation,

$$\delta F_A = \left[K_{AB} \right] \delta r_B \quad (3)$$

δF_A denotes for the reaction force on atom A by displacing δr_B of atom B. $\left[K_{AB} \right]$ is the interatomic force constant matrix. It has three eigenvalues λ_i^{AB} and three eigenvector $\hat{v}_i^{AB}$ (i = 1, 2, 3), with unitary norm. The bond stretching force constant for bond A-B， K_{AB} is obtained from equation (4)

$$K_{AB} = \sum_{i=1}^{3} \lambda_i^{AB} \left| \hat{u}^{AB} \cdot \hat{v}_i^{AB} \right| \quad (4)$$

$\hat{u}^{AB}$ is the unit vector pointing from A to B. It is notable that $K_{AB} = 2K_r$ (K_r is the bond stretching force constant in equation 1).

To determine the angle-bending force constant k_θ for angle $\angle$ABC, we considered the bonds A-B and C-B that have unitary vector $\hat{u}^{AB}$ and $\hat{u}^{CB}$. Eigenvalues λ_i^{AB} and λ_i^{CB} , and eigenvectors $\hat{v}_i^{AB}$ and $\hat{v}_i^{CB}$ (i = 1, 2, 3) determined from their interatomic matrices $\left[K_{AB} \right]$ and $\left[K_{CB} \right]$. Defined $\hat{u}_N$ to be a unit vector perpendicular to the ABC plane:

$$\hat{u}_N = \frac{\hat{u}^{CB} \times \hat{u}^{AB}}{\left|\hat{u}^{CB} \times \hat{u}^{AB}\right|} \qquad (5)$$

The unit vectors perpendicular to the bonds A-B and C-B on the plane ABC are given by equation 6 and 7,

$$\hat{u}^{PA} = \hat{u}^{AB} \times \hat{u}_N \qquad (6)$$

$$\hat{u}^{PC} = \hat{u}^{CB} \times \hat{u}_N \qquad (7)$$

They represent the direction of small displacements of atoms A and C that result from opening or closing the angle <ABC.

$\mathrm{R_{AB}}$ and $\mathrm{R_{CB}}$ are assigned to be the length of the bonds, A-B and C-B. The angle-bending force constant can be derived as:

$$\frac{1}{k_\theta} = \frac{1}{\mathrm{R_{AB}^2} \sum_{i=1}^{3} \lambda_i^{AB} \left|\hat{u}^{PA} \cdot \hat{v}_i^{AB}\right|} + \frac{1}{\mathrm{R_{CB}^2} \sum_{i=1}^{3} \lambda_i^{CB} \left|\hat{u}^{PC} \cdot \hat{v}_i^{CB}\right|} \qquad (8)$$

Here, $k_\theta = 2K_\theta$ (K_θ is the angle-bending force constant in equation 1).

Calculations of three models as shown in Figure 1 were performed as described above using the MTK++ program.[6] Neglecting the dihedral parameters is a common procedure for this symmetric geometry of the metal coordination center.[7]

Atomic single charge calculations. Large manganese cluster models containing all atoms of a bound residue were constructed to keep the native crystallographic geometry. These models (Model II) were capped with acetyl (ACE) and N-methylamino (NME) residues (Figure S4). Sampling was conducted by the Merz-Singh-Kollman (MK)[8] while the restrained electrostatic potential (RESP) method was used to derive atom-centered partial charges. Electrostatic potential (ESP) charge fitting was performed by QM computations using Gaussian 09, with keywords

WB97XD/6-31G** for C, H, O and N, but LANL2DZ for Mn. A Van der Waals radius of 1.69 Å was assigned to the manganese center.[9] This task was conducted based on the RESP fitting protocol implemented in the MTK++ program. This protocol restrains the backbone heavy atoms (CA, N, C, O) to those values found in the AMBER parm94 force field and has given the most impressive performance in previous reports.

Calculated vibrational frequencies. To check the quality of the force field parameters, we compared the Quantum Mechanics (QM) and Molecular Mechanics (MM) vibrational frequencies. The three models (Model II) illustrated in Figure 2 were used to perform the structural optimization and frequency analysis at the WB97XD/6-31G** (C, H, O and N), LANL2DZ (Mn) level by QM. The Multiwfn program[10] was used to derive the vibrational frequencies from the results of the frequency analysis. At the MM level, all of the structural optimizations were performed by the Nucleic acid builder (NAB) module of AmberTools 13.[11] The Newton-Raphson method was employed to carry out the energy minimization. The convergence criterion was set to 10^{-12} kcal/(mol·Å). The distance cutoff of nonbonded interactions was set to 999 Å. The vibrational frequencies of MM were produced by NAB with the ff99SB[12]parameters in normal-mode analysis.

MD simulations. All MD simulations were performed with the AMBER12 package.[11] MD results were analyzed with the PTRAJ module of the AMBER package. Hydrogen bonds (H-bond) were also assigned using the PTRAJ on the basis of the following criteria: the distance between the proton donor and acceptor atoms was ≤3.5 Å, and the angle formed by the donor, hydrogen, and acceptor was ≥120°.[13] The force field parameters obtained in our study were applied to the molecular dynamics (MD) simulations of three systems in explicit solvents (Figure S2). The RESP

fitting charges were only applied to the Mn (II)-Mn (II) center of three systems indicated in Figure 2. The partial charges from the force field ff99SB set were used to assign the rest of parts on three systems. A bond was created to mimic the coordination interaction between the manganese ion and manganese-related atom both on the protein side and ligand side. These systems were soaked in a rectangular box of TIP3P water[14] with the smallest distance between the protein surface and cell boundary set to 10 Å. An appropriate number of counterions were added to neutralize the global charge of the entire system. For each system, we performed three individual 50ns MD at different seeds for production phase without any restraint was performed. This was preceded by 2000 steps energy minimization with a weak positional restraint to eliminate unfavorable contacts, 500 ps slowly heating in the canonical ensemble (NVT) from 0 K to 300 K and 500 ps of density equilibration to adjust the solvent density under 1 atm pressure in the isothermal–isobaric ensemble (NPT), and followed by a 5 ns constant pressure equilibration which was performed unrestrained at 300 K. All MD simulations were carried out using a Langevin thermostat[15] with a collision frequency of 2.0 ps^{-1} dynamics for temperature control. Constant pressure was controlled with an average pressure of 1 atm using a Berendsen barastat.[16] The time step was set to 2 fs. The periodic boundary condition was enabled during MD simulation. The particle mesh ewald (PME) method[17] was used to handle the long-range electrostatic interactions. The distance cutoff for the real-space nonbond interactions was set to 12 Å. All bonds with hydrogen atoms were constrained using the SHAKE algorithm.[18] The three individual 50ns MD trajectories for each system was recorded every 10 ps for subsequent analysis.

Binding free energy calculation and spectrum of free energy decomposition. The binding free energy for both systems was estimated by the Molecular Mechanics-Poisson-Boltzmann Surface

Area (MM-PBSA) approach as implemented in AMBER12 using the same force field parameters as described above. MM-PBSA calculations were performed on 1000 snapshots exacted from 40~50 ns production trajectories with a time interval of 10 ps. The binding free energy (ΔG_{bind}) of each system was evaluated as follows: $\Delta G_{bind} = \Delta H - T\Delta S \approx \Delta E_{MM} + \Delta G_{solv} - T\Delta S$

(1)

$$\Delta E_{MM} = \Delta E_{\text{int}} + \Delta E_{ele} + \Delta E_{vdW} \quad (2)$$

$$\Delta G_{solv} = \Delta G_{PB} + \Delta G_{SA} \quad (3)$$

$$\Delta G_{SA} = \gamma SASA + \beta \quad (4)$$

The molecular mechanics energy, ΔE_{MM}, is the gas-phase interaction energy between the receptor and the ligand including the internal energies (bond, angle, and dihedral energies; ΔE_{int}), the electrostatic energies (ΔE_{ele}) and the van der Waals energies (ΔE_{vdW}). ΔG_{solv}, this sum of solvation energy can be divided into the polar solvation energy and the nonpolar solvation energy. The polar solvation energy ΔG_{PB} was evaluated by solving the linearized MM-PBSA equation in a continuum solvent model. The nonpolar solvation energy ΔG_{SA} was estimated by linearly relating it to the solvent-accessible surface area ($SASA$), according to equation 4.[19] The surface tension proportionality coefficient γ and the offset parameter β were set to 0.00542 kcal/(mol ·Å^2) and 0.92 kcal/mol. The solvent probe radius 1.4 Å was applied to derive $SASA$ values.[20] The solute dielectric constant was set to 1 and 80 as the external counterpart of the solute was applied. $-T\Delta S$ is the change of the conformational entropy upon ligand binding, which was not considered here because our aim is to compare the binding affinity of three ligands in our three systems.

In order to estimate the contribution of key residues on the ligand binding, the protein-ligand

interaction spectrum of each complex was decomposed based on a per-residue method[21] using the MM-PBSA decomposition analysis by the mmpbsa.py module in AMBER12. The energy contribution for each ligand-residue pair has four components as shown in equation 5:

$$\Delta G_{ligand-residue} = \Delta E_{Vdw} + \Delta E_{ele} + \Delta G_{PB} + \Delta G_{CAVITY} \qquad (5)$$

Where ΔE_{Vdw} and ΔE_{ele} represent the non-bonded van der Waals contribution and the electrostatic energy. ΔG_{PB} and ΔG_{CAVITY} stand for the electrostatic contribution and the nonpolar contribution to the solvation free energy, respectively. ΔG_{PB} was calculated by the PB model, and ΔG_{CAVITY} was obtained by an empirical model based on the corresponding $SASA$. The decomposition energies for each residue in the complex are further broken down into backbone, sidechain, and total energy contributions. All energy components in equation 5 were calculated using the same snapshots as the binding free energy calculation.

Computational alanine scanning mutagenesis. The computational alanine scanning mutagenesis (ASM) protocol has been widely used in structure-based drug design and protein engineering for evaluating the contributions of individual residue side chains to protein−protein or protein−ligand binding free energy and understanding the structural and energetic characteristics of the hot-spots. It has been shown to be an effective and reliable method, and can now be applied with an accuracy of 1 kcal/mol. ASM is capable of anticipating the experimental results of site-directed mutagenesis and achieves an overall success rate of 80% and an 82% success rate in residues for which alanine mutation causes an increase in the binding free energy > 2.0 kcal/mol (warm- and hot-spots).[22] To further evaluate the impact of mutational effects on the ligands binding in our three systems, ASM was applied to estimate the relative binding free energy of different PP5c mutants to the ligands. The binding free energies for the complex and for the alanine mutants were calculated using the

MM-PBSA method. We used the same 1000 snapshots taken from the production phase MD trajectory for performing the alanine scanning. The alanine mutant trajectory was initially generated from the wild type molecular dynamics trajectory by truncating the side chains of the mutated residue at Cγ replacing them with a hydrogen atom and setting the Cβ-H direction to that of the former Cβ-Cγ. The relative binding free energy is the free energy difference between the wild-type and alanine mutants and is defined as:

$$\Delta\Delta G_{binding} = \Delta G_{binding-mu\tan t} - \Delta G_{binding-wildtype}$$

Where $\Delta G_{binding-mu\tan t}$, $\Delta G_{binding-wildtype}$ represent the binding free energies of wild type and mutant complexes, respectively. A positive $\Delta\Delta G_{binding}$ indicates that ligand binding to the wild-type protein is more favorable.

Site-directed mutagenesis. The sequence encoding the ORF of Human PP5 (HuPP5) was isolated from the liver cancer cell line SMMC-7721 by PCR using primers that introduced a Nde I site and 6xHis tag at the forward one, and a Xho I site at the reverse one. The PCR products were cloned into pCR2.1 (Invitrogen, Carlsbad, CA, USA) and transformed into DH5α (TaKaRa, Dalian, China) and then sequenced (AuGCT, Inc., Beijing, China). The mutants were generated using the QuikChange® site-directed mutagenesis kit (Stratagene, La Jolla, CA, USA) following the instruction manual. The mutagenic primers were designed based on the sequence of wild-type HuPP5 and are listed in Table S5. The mutants were sequenced to confirm the presence of the desired mutations and the lack of undesired mutations.

Protein expression and purification. The wild-type and mutants HuPP5 were then cloned into the corresponding sites of pET43.1b (Novagen, Madison, WI, USA) and transformed into *Escherichia coli* BL21(DE3)pLysS (Novagen, Madison, WI, USA). Proteins were expressed in LB

(Luria-Bertani) broth (containing 2 mM $MnCl_2$, 100 μg/ml ampicillin and 34 μg/ml chloramphenicol) and induced with 0.1 mM IPTG (isopropyl β-thiogalactoside) for 24 h at 18 ℃. Cells were harvested by centrifugation (8,000 g for 10 min at 4 ℃) and were lysed by sonication in buffer A (20 mM Tris-HCl, pH=8.0, 4 mM $MnCl_2$, 300 mM NaCl) containing 20 mM imidazole, 0.1% β-mercaptoethanol, 1.0 mg/ml lysozyme and 1 mM PMSF. After centrifugation for 20 min at 20,000 g at 4 ℃, the supernatant was loaded to a buffer A pre-equilibrated Ni-NTA affinity column (Transgen, Beijing, China) to bind the 6xHis tagged protein. The reduction of non-specific interactions between the extract proteins and the matrix was achieved by washing the Ni-NTA resins with buffer B (20 mM Tris-HCl, pH=8.0, 300 mM NaCl, 1 mM EGTA, 0.1% β-mercaptoethanol, 4 mM $MnCl_2$) containing 20 mM imidazole. Elution was then conducted using 150 mM imidazole in buffer B. The eluted HuPP5 fractions were pooled and dialyzed against buffer C (buffer B + 50% glycerol) overnight. Dialyzed samples were subjected to 12% SDS-PAGE and stained with Coomassie blue. Protein concentrations were determined using a BCA Protein Assay Kit (Pierce, Thermo Scientific, Rockford, IL, USA).

Enzymatic assays and inhibition studies. Phosphatase activity was measured using p-NPP (p-nitrophenyl phosphate) as substrate. Assays were performed in a 200 μL reaction containing 500 ng protein and assay buffer (20 mM p-NPP in 20 mM Tris (pH 7.4), 100 μM arachidonic acid, 1 mM EGTA, 0.1% β-mercaptoethanol and 0.1% ethanol). The reaction was initiated by adding the p-NPP and incubated at 35 ℃ for 10 min. Control reactions were assayed without enzyme. Assays were terminated by addition of 200 μL of 5 N NaOH. Sample absorbance was measured at A_{410} using the TECANTM Infinite® 200 PRO multimode micro-plate reader after subtracting the appropriate background. Kinetic parameters were determined by using the Michaelis-Menten

plot analysis of data obtained under the assay conditions as mentioned above with the addition of different concentrations of p-NPP (1, 2, 5, 10, 20, 40, and 80 mM).

Inhibition assays were carried out by adding compounds to the assay mixture 5 min before starting the reaction with the addition of p-NPP as described above. Compounds were dissolved in DMSO (dimethyl sulphoxide) to obtain 1 M stock solutions and diluted to desired concentrations with assay buffer before use. A non-enzyme reaction was used as the background control, while a non-compound reaction was taken as the full-activity control. The inhibition ratio was calculated as the percentage of A_{410} values of the inhibition assay reaction divided by the full-activity control, having subtracted the background control A_{410} value for both. The IC_{50} values were obtained using GraphPad Prism 5.0.

References

1. Bertini, I., Calderone, V., Fragai, M., Luchinat, C. & Talluri, E. Structural basis of serine/threonine phosphatase inhibition by the archetypal small molecules cantharidin and norcantharidin. *J Med Chem* **52**, 4838-4843 (2009).
2. M. J. Frisch, G. W. T. *et al.* Gaussian 09, Revision B.01. *Gaussian, Inc., Wallingford CT, 2010..* , (2010).
3. Zhang, H., Ma, Y., Liu, K. & Yu, J. Theoretical studies on the reaction mechanism of PP1 and the effects of different oxidation states of the Mn–Mn center on the mechanism. J Biol Inorg Chem **18**, 451-459 (2013).
4. Chai, J. D. & Head-Gordon, M. Long-range corrected hybrid density functionals with damped atom-atom dispersion corrections. *Phys Chem Chem Phys* **10**, 6615-6620 (2008).
5. Cornell, W. D. *et al.* A Second Generation Force Field for the Simulation of Proteins, Nucleic Acids, and Organic Molecules. *J Am Chem Soc* **117**, 5179 (1995).
6. Peters, M. B. *et al.* Structural Survey of Zinc Containing Proteins and the Development of the Zinc AMBER Force Field (ZAFF). *J Chem Theory Comput* **6**, 2935-2947 (2010).
7. Hoops, S. C., Anderson, K. W. & Merz, K. M. Force field design for metalloproteins. *J Am Chem Soc* **113**, 8262 (1991).
8. Singh, U. C. An approach to computing electrostatic charges for molecules. *J Comput Chem* **5**, 129 (1984).
9. M. Bradbrook, G. X-Ray and molecular dynamics studies of concanavalin-A glucoside and mannoside complexes Relating structure to thermodynamics of binding. *J Chem Soc Faraday Trans* **94**, 1603 (1998).
10. Lu, T. Multiwfn: A multifunctional wavefunction analyzer. *Journal of Computational Chemistry* **33**, 580 (2012).
11. D.A. Case, T. A. D. *et al.* AMBER 12, University of California, San Francisco , (2012).
12. Hornak, V. Comparison of multiple Amber force fields and development of improved protein backbone parameters. *Proteins* **65**, 712 (2006).
13. Aruksakunwong, O. *et al.* On the Lower Susceptibility of Oseltamivir to Influenza Neuraminidase Subtype N1 than Those in N2 and N9. *Biophys J* **92**, 798 (2007).
14. Jorgensen, W. L. Theoretical studies of medium effects on conformational equilibria. *J Phys Chem* **87**, 5304 (1983).
15. Loncharich, R. J. Langevin dynamics of peptides: The frictional dependence of isomerization rates of N-acetylalanyl-N′-methylamide. *Biopolymers* **32**, 523 (1992).
16. Berendsen, H. J. C., Postma, J. P. M., van Gunsteren, W. F., DiNola, A. & Haak, J. R. Molecular dynamics with coupling to an external bath. *J Chem Phys* **81**, 3684-3690 (1984).
17. Essmann, U. *et al.* A smooth particle mesh Ewald method. *J Chem Phys* **103**, 8577-8593 (1995).
18. Ryckaert, J., Ciccotti, G. & Berendsen, H. J. C. Numerical integration of the cartesian equations of motion of a system with constraints: molecular dynamics of n-alkanes. *J Comput Phys* **23**, 327 (1977).
19. Weiser, J. Approximate atomic surfaces from linear combinations of pairwise overlaps (LCPO). *J Comput Chem* **20**, 217 (1999).
20. Sitkoff, D., Sharp, K. A. & Honig, B. Accurate Calculation of Hydration Free Energies Using Macroscopic Solvent Models. *J Phys Chem* **98**, 1978 (1994).
21. Gohlke, H., Kiel, C. & Case, D. A. Insights into Protein–Protein Binding by Binding Free Energy Calculation and Free Energy Decomposition for the Ras–Raf and Ras–RalGDS Complexes. *J Mol Biol* **330**, 891 (2003).
22. Moreira, I. S. Computational alanine scanning mutagenesis—An improved methodological approach. *J*

Comput Chem **28**, 644 (2007).
